# Supplementary material for: Modelling to infer the role of animals in gambiense human African trypanosomiasis transmission and elimination in the DRC
Source: PLoS Negl Trop Dis. 2022 Jul 11;16(7):e0010599. doi: 10.1371/journal.pntd.0010599 (PMC9302778; doi:10.1371/journal.pntd.0010599)
Supplement: S1 Text — More detailed description of materials and methods, and additional results and figures. Fig B: Bivariate choropleth showing support for the models with or without animals contributing to transmission and the difference in the probability of achieving EoT to humans by 2030 from these two models. Bivariate choropleth showing support for the models with or without animals contributing to transmission and the difference in the probability (Pd) of achieving EoT to humans by 2030 from these two models (“High” is more than 10% difference, “Medium” is 5-10% difference, and “Low” is less than 5% difference). Shapefiles used to produce these map were provided by Nicole Hoff and Cyrus Sinai under a CC-BY licence (current versions can be found at https://data.humdata.org/dataset/drc-health-data). Fig D: The percentage of health zones studied expected to have reached elimination of transmission (EoT) against year, for the models without animal transmission, with animal transmission, and an ensemble of these two models. The percentage of health zones studied expected to have reached elimination of transmission (EoT) against year, for the models without animal transmission, with animal transmission, and an ensemble of these two models. Active screening post-2016 was assumed to be at the mean level observed 2012–2016, and passive screening continued at the 2016 level of effectiveness. Vector control (VC) was either simulated in none (solid lines), all (dashed lines), or a subset of health zones in which the probability of reaching EoT by 2030 without VC was less than 0.9 (dotted lines; using this cut-off measure, VC was simulated in 77% of health zones in the ensemble model, 76% of health zones in the model without animal transmission, and in 79% of health zones in the model with animal transmission). Fig E: Mean prevalence of infection within year as a percentage of the population. Mean prevalence of infection within year as a percentage of the population. Human cases are consider [file pntd.0010599.s001.pdf]

Supplementary Information: Modelling to infer the role of animals in *gambiense* human African trypanosomiasis transmission and elimination in the DRC S1 Text: Additional methods and results

Ronald E. Crump[1,2◑*], Ching-I Huang[1,2◑], Simon E. F. Spencer[1,3], Paul E. Brown[1,2], Chansy Shampa[4], Erick Mwamba Miaka[4], and Kat S. Rock[1,2]

[1]Zeeman Institute for Systems Biology and Infectious Disease Epidemiology Research, The University of Warwick, Coventry, U.K.
[2]Mathematics Institute, The University of Warwick, Coventry, U.K.
[3]The Department of Statistics, The University of Warwick, Coventry, U.K.
[4]Programme National de Lutte contre la Trypanosomiase Humaine Africaine (PNLTHA), Kinshasa, D.R.C.

July 6, 2022

◑These authors contributed equally to this work.
* Corresponding author: r.e.crump@warwick.ac.uk

Contents

S1.1 Data 2

S1.2 The Compartmental gHAT Model 4
S1.2.1 Model parameterisation 6

S1.3 Modelling passive detection and its improvement 8

S1.4 Modelling vector control 10

S1.5 Fitting 12
S1.5.1 Running the models 12
S1.5.2 Likelihood 12
S1.5.3 Imputation of missing numbers screened information 13

S1.6 Host-specific contributions to transmission 15

S1.7 Model comparison 17

S1.8 Additional results 18
S1.8.1 Ensemble model 18
S1.8.2 Prevalence of infection 19

List of Figures

A Impact of tiny targets on tsetse density 11
B Bivariate choropleth showing support for the models with or without animals contributing to transmission and the difference in the probability of achieving EoT to humans by 2030 from these two models 18

C Maps of the probability that end of transmission in humans is achieved by 2030 for the models with and without animal transmission, and an ensemble model of these. 19
D The percentage of health zones studied expected to have reached elimination of transmission (EoT) against year, for the models without animal transmission, with animal transmission, and an ensemble of these two models. 20
E Mean prevalence of infection within year as a percentage of the population. 21

List of Tables

A Number of HAT Atlas records with different combinations of former province, health zone and health area recorded. 2
B Model parameterisation (fixed parameters) 6
C Model parameterisation (fitted parameters) 7
D Parameterisation of passive detection improvement 9
E Imputation of negative active screening results 14
F Next generation matrix; non-zero elements of the transmission matrix, $\mathbf{T}$ 15
G Next generation matrix; non-zero elements of the transition matrix, Σ 16

S1.1 Data

This section outlines the methods used to convert available data into a format suitable for model fitting and is the same as described in Crump et al. [7].

HAT Atlas data The HAT Atlas data for the DRC were provided in a spreadsheet format. Records were annually aggregated gHAT case records, aggregated by year, surveillance type and location as defined by multiple fields. There were 117,573 rows in this file; of which 111,408 had an entry in the geolocation (longitude and latitude) fields.

Passive surveillance records with missing or zero case numbers; and active surveillance records with both missing or zero numbers screened and missing or zero case numbers were dropped from the dataset.

This left 111,454 records (105,979 with filled geolocation fields). These records were associated with 23,424 unique combinations of former province, health zone, health area, location and territory identifiers; 20,423 of which had geolocation information and 3,001 did not. We will refer to these 24,424 geographical records as gHAT locations in this document.

Table A: Number of HAT Atlas records with different combinations of former province, health zone and health area recorded.

Recorded region identifiers:			Number
Former province	Health zone	Health area	
✓	✓	✓	106823
✓	✓	✓	391
✓	✓		3001
✓		✓	14
✓			1225

DRC Shapefile A recent shapefile for the DRC was provided by UCLA (Personal communication). The shapefile contains health zones (an organisational unit with a typical population size around 100,000) across the DRC; health areas (nested within health zones, these areas are typically home to around 10,000 people) for the former province of Bandundu and part of Equateur and Haut Lomami, and post–2015 province identifiers. Former province was added to these records (post–2015 provinces being nested within former province).

Additional geographic information The following geographical information was obtained from the Humanitarian Data Exchange [24]:

- a health zone shapefile from the United Nations Office for the Coordination of Humanitarian Affairs (OCHA);
- an OCHA file of geolocations of localities; and
- a file of geolocations of health facilities from the Global Healthsite Mapping Project.

These data were used to assist in matching and locating the gHAT data, by providing alternative spellings of names and potentially geolocations for non-geolocated gHAT locations. The locality and health facility lists were concatenated, and this enlarged locality set and the OCHA health zone map were assigned geographical identifiers as per our shapefile of choice.

Matching HAT Atlas records to the DRC shapefile Geographical identifiers associated with the HAT Atlas/gHAT location and geographical data were sanitised to assist with matching. This involved removing diacritical marks, conversion to lowercase, collapsing whitespace within identifiers to a single space, removal of leading and trailing whitespace, converting from roman to arabic numerals, removing leading m, n or g from words where they were followed by a consonant, removing leading t from words when followed by an s, and collapsing words into a single string. In addition some specific manual edits were performed during the process as they became apparent.

Matching was then applied sequentially to the gHAT locations; such that once a match had been achieved for any given gHAT location it did not act as input to subsequent steps.

1. gHAT locations with known former province (FP), health zone (HZ), health area (HA) and geolocation were located on the UCLA and OCHA shapefiles. If the FP, HZ and HA matched the values for either the UCLA or OCHA shapefiles at that point, a match was judged to have occurred and the geolocation was accepted. This matched 3,579 of the gHAT locations.
2. gHAT locations with known former province (FP), health zone (HZ) and geolocation were located on the UCLA and OCHA shapefiles. If the FP and HZ matched the values for either the UCLA or OCHA shapefiles at that point, a match was judged to have occurred and the geolocation was accepted. This matched 13,413 of the gHAT locations. *The 16,992 gHAT locations matched in these two steps accounted for 97,520 of the HAT records (87.5%).*
3. Where a gHAT location was associated with a recent active screening event (defined as an active screening record in or after 2012 with a number screened greater than 10), the geolocation was accepted. *This matched a further 454 of the gHAT locations to give a total of 100,747 gHAT records matched (90.4%).*
4. Matching to the locality information:
   (a) if health zone and location identifier match, the locality's geolocation was assigned to the gHAT location. *Matching 44 gHAT locations, giving a total of 100,811 gHAT records matched (90.5%).*
   (b) if former province and location identifier match, the locality's geolocation was assigned to the gHAT location. *This matched 331 gHAT locations, giving a total of 101,508 gHAT records matched (91.1%).*
5. Matching to shapefile by geographic identifiers only:
   (a) Former province, health zone and health area all match. *Matched 523 gHAT locations, giving a total of 102,349 gHAT records matched (91.8%).*
   (b) Former province and health zone match. *Matched 3636 gHAT locations, giving a total of 110,021 gHAT records matched (98.7%).*
   (c) Former province and OCHA shapefile health zone name match. *Matched 74 gHAT locations, giving a total of 110,126 gHAT records matched (98.8%).*
   (d) Former province and health area match. *Matched 60 gHAT locations, giving a total of 110,195 gHAT records matched (98.9%).*
   (e) Health zone name match. *Matched 10 gHAT locations, giving a total of 110,210 gHAT records matched (98.9%).*

S1.2 The Compartmental gHAT Model

The gHAT models we considered in this study were variants "Model 4" (without animal transmission) and "Model 7" (with animal transmission) of the Warwick model previously presented in the literature [7, 16, 17, 12, 18]. gHAT infections among hosts are described by equation (S1.2.1) for the model with animal transmission. The model without animal transmission uses the same equations for human hosts and vectors, but not the animal host equations. Human hosts are modelled by a susceptible-exposed-infectious-infectious-recovered-susceptible (SEIIRS) model with two infectious compartments, stage 1 disease, I_{1H}, and stage 2 disease, I_{2H}. Animal hosts that contribute to transmission are described by a susceptible-exposed-infectious model, with infected animals remaining infectious for life.

Vectors are modelled by using compartments to appropriately model tsetse when used in a host-vector model with disease [17]. Pupal stage tsetse, P_V, emerge into unfed susceptible adults, S_V, and following a blood-meal become either exposed, E_V, or have reduced susceptibility to the *Trypanosoma brucei gambiense* parasites, G_V - this effect is known as the teneral phenomenon. Following an infection, tsetse have an extrinsic incubation period (EIP) before becoming onwardly infectious. To incorporate a more realistic EIP distribution, there are three exposed classes, E_{1V}, E_{2V}, E_{3V}, which results in a gamma-distributed EIP (rather than an exponential EIP with a single exposed class).

In order to reduce the dimensionality of our ODE system (by one), the vector equations are non-dimensionalised using the scaling N_H/N_V, where N_H is the total human population, and N_V is the tsetse population size. This results in a new non-dimensionalised parameter, m_{eff}, which is $\frac{p_H N_V}{N_H}$ appearing in host equations (p_H is the probability of a human being infected by a single infectious bloodmeal) and is referred to as the *effective vector density*.

The proportion of tsetse bites taken on low-risk and high-risk humans are f_1 and f_4, depending on the relative availability/attractiveness and the relative abundance of two risk groups. High-risk humans are assumed to be r-fold more likely to receive bites, i.e. $s_1 = 1$ and $s_4 = r$. Therefore, f_i's can be calculated using $f_i = \frac{s_i N_{Hi}}{\sum_j s_j N_{Hj}}$.

$$
\text{Humans}\quad\left\{\begin{aligned}
\frac{\mathrm{d}S_{Hi}}{\mathrm{d}t} &= \mu_H N_{Hi} + \omega_H R_{Hi} - \alpha m_{\text{eff}} f_i \frac{S_{Hi}}{N_{Hi}} I_V - \mu_H S_{Hi}\\
\frac{\mathrm{d}E_{Hi}}{\mathrm{d}t} &= \alpha m_{\text{eff}} f_i \frac{S_{Hi}}{N_{Hi}} I_V - (\sigma_H + \mu_H) E_{Hi}\\
\frac{\mathrm{d}I_{1Hi}}{\mathrm{d}t} &= \sigma_H E_{Hi} - (\varphi_H + \eta_H(Y) + \mu_H) I_{1Hi}\\
\frac{\mathrm{d}I_{2Hi}}{\mathrm{d}t} &= \varphi_H I_{1Hi} - (\gamma_H(Y) + \mu_H) I_{2Hi}\\
\frac{\mathrm{d}R_{Hi}}{\mathrm{d}t} &= \eta_H(Y) I_{1Hi} + \gamma_H(Y) I_{2Hi} - (\omega_H + \mu_H) R_{Hi}
\end{aligned}\right.
$$

$$
\text{Animals}\quad\left\{\begin{aligned}
\frac{\mathrm{d}S_A}{\mathrm{d}t} &= \mu_A N_A - \alpha m_{\text{eff}} f_A \frac{S_A}{N_A} I_V - \mu_A S_A\\
\frac{\mathrm{d}E_A}{\mathrm{d}t} &= \alpha m_{\text{eff}} f_A \frac{S_A}{N_A} I_V - (\sigma_A + \mu_A) E_A\\
\frac{\mathrm{d}I_A}{\mathrm{d}t} &= \sigma_A E_A - \mu_A I_A
\end{aligned}\right.
$$

$$
\text{Tsetse}\quad\left\{\begin{aligned}
\frac{\mathrm{d}P_V}{\mathrm{d}t} &= B_V N_H - (\xi_V + \tfrac{P_V}{K}) P_V\\
\frac{\mathrm{d}S_V}{\mathrm{d}t} &= \xi_V \mathbb{P}(\text{survive pupal stage}) P_V - \alpha S_V - \mu_V S_V\\
\frac{\mathrm{d}E_{1V}}{\mathrm{d}t} &= \alpha\big(1 - f_T(t)\big) p_V \big(\textstyle\sum_i f_i \dfrac{(I_{1Hi} + I_{2Hi})}{N_{Hi}} + f_A \dfrac{I_A}{N_A}\big)(S_V + \varepsilon G_V)\\
&\qquad -(3\sigma_V + \mu_V + \alpha f_T(t)) E_{1V}\\
\frac{\mathrm{d}E_{2V}}{\mathrm{d}t} &= 3\sigma_V E_{1V} - (3\sigma_V + \mu_V + \alpha f_T(t)) E_{2V}\\
\frac{\mathrm{d}E_{3V}}{\mathrm{d}t} &= 3\sigma_V E_{2V} - (3\sigma_V + \mu_V + \alpha f_T(t)) E_{3V}\\
\frac{\mathrm{d}I_V}{\mathrm{d}t} &= 3\sigma_V E_{3V} - (\mu_V + \alpha f_T(t)) I_V\\
\frac{\mathrm{d}G_V}{\mathrm{d}t} &= \alpha(1 - f_T(t))\Big(1 - p_V\big(\textstyle\sum_i f_i \dfrac{(I_{1Hi} + I_{2Hi})}{N_{Hi}} + f_A \dfrac{I_A}{N_A}\big)\Big) S_V\\
&\qquad -\alpha\Big(f_T(t) + \big(1 - f_T(t)\big) p_V \varepsilon \big(\textstyle\sum_i f_i \dfrac{(I_{1Hi} + I_{2Hi})}{N_{Hi}} + f_A \dfrac{I_A}{N_A}\big)\Big) G_V\\
&\qquad\quad -\mu_V G_V
\end{aligned}\right.
\tag{S1.2.1}
$$

S1.2.1 Model parameterisation

Table B: **Model parameterisation (fixed parameters).** Notation, a brief description, and the values used for fixed parameters.

Notation	Description	Value	
$N_H{}^*$	Total human population size in 2015	Fixed for each health zone	[14]
μ_H	Natural human mortality rate	5.4795×10^{-5} days^{-1}	[20]
B_H	Total human birth rate	$= \mu_H N_H$	
σ_H	Human incubation rate	0.0833 days^{-1}	[19]
φ_H	Stage 1 to 2 progression rate	0.0019 days^{-1}	[2, 4]
ω_H	Recovery rate or waning-immunity rate	0.006 days^{-1}	[13]
Sens	Active screening diagnostic sensitivity	0.91	[3]
$B_V{}^\dagger$	Tsetse birth rate (per capita rate of depositing new pupae)	0.0505 days^{-1}	[17]
ξ_V	Rate of pupal development to adult flies	0.037 days $^{-1}$	[17]
$K^\ddagger$	Pupal carrying capacity	$= 111.09 N_H$	[17]
$\mathbb{P}$(pupating)	Probability of a pupa surviving to emerge as an adult fly	0.75	[17]
μ_V	Tsetse mortality rate	0.03 days^{-1}	[19]
σ_V	Tsetse incubation rate	0.034 days^{-1}	[8, 15]
α	Tsetse bite rate	0.333 days^{-1}	[25]
p_V	Probability of tsetse infection per single infective bite	0.065	[19]
ε	Reduced susceptibility factor for non-teneral (previously fed) flies	0.05	[16]
f_H	Proportion of blood-meals on humans	0.09	[5]
η_H^{pre}	Treatment rate from stage 1, pre-1998	0	Assumed
$\text{disp}_{\text{act}}{}^\S$	Overdispersion parameter for active detection	4×10^{-4}	[7]
$\text{disp}_{\text{pass}}{}^\S$	Overdispersion parameter for passive detection	2.8×10^{-5}	[7]
**Parameters specific to the model with animal transmission...**			
μ_A	Natural animal mortality rate	0.0014 days^{-1}	Assumed
σ_A	Animal incubation rate	0.0833 days^{-1}	[19]

*The model is internally scaled such that the population size in all years corresponds to the population in 2015 (outputs are back-transformed to reflect an assumed annual population growth rate of 3% across the DRC).
†The value of B_V was chosen to maintain constant population size in the absence of vector control interventions.
‡The value of K was chosen to reflect the observed bounce back rate.
§Over-dispersion values were chosen based on a comparison of the median of the distributions of log posterior probability from MCMC runs with ρ fixed at a range of values for two example health zones under the model without animal transmission [7].

Table C: **Model parameterisation (fitted parameters).** Notation, brief description, and information on the prior distributions for fitted parameters.

Notation	Description	Prior distribution*	Percentiles of prior distribution [2.5, 50 & 97.5%]	Unit
R_0	Basic reproduction number (NGM approach)	$1 + \text{Exp}(10)$	[1.003, 1.069, 1.369]	-
r	Relative bites taken on high-risk humans	$1 + \Gamma(3.68, 1.09)$	[2.015, 4.654, 10.028]	-
k_1	Proportion of low-risk people	$\text{B}(16.97, 3.23)$	[0.6564, 0.8514, 0.9609]	-
$\eta_H^{\text{post}\,\dagger}$	Treatment rate from stage 1, 1998 onwards	$\Gamma(3.54, 5.32 \times 10^{-5})$	[4.59, 17.1, 42.9]$\times 10^{-5}$	days^{-1}
$\gamma_H^{\text{post}\,\dagger}$	Combined treatment and disease-induced death rate from stage 2, 1998 onwards	$\Gamma(2.45, 0.00192)$	[7.59, 40.7, 121]$\times 10^{-4}$	days^{-1}
$b_{\gamma_H^{\text{pre}}}{}^{\P}$	Relative treatment/death rate from stage 2 factor, pre-1998	$\text{B}(1, 1)$	[0.025, 0.500, 0.975]	-
Spec	Active screening diagnostic specificity	$0.998 + (1 - 0.998)\,\text{B}(7.23, 2.41)$	[0.9989, 0.9995, 0.9999]	-
u	Proportion of stage 2 cases reported from passive screening	$\text{B}(20, 40)$	[0.2208, 0.3315, 0.4564]	-
$d_{\text{change}}{}^{\ddagger}$	Midpoint year for passive improvement	$2000 + (2017 - 2000)\,\text{B}(5, 6)$	[2003.2, 2007.7, 2012.5]	year
$\eta_{H_{\text{amp}}}{}^{\S}$	Relative improvement in passive screening stage 1 detection rate	$\Gamma(2.013, 1.049)$	[0.258, 1.775, 5.870]	-
$\gamma_{H_{\text{amp}}}{}^{\S}$	Relative improvement in passive screening stage 2 detection rate	$\Gamma(1.001, 5)$	[0.127, 3.471, 18.455]	-
$d_{\text{steep}}{}^{\S}$	Speed of improvement in passive screening detection rate	$\Gamma(39.57, 0.0270)$	[0.761, 1.058, 1.424]	years^{-1}
**Parameters specific to the model with animal transmission...**				
f_A	Proportion of blood meals on reservoir animals	Flat over range 0–1	$[0.025, 0.5, 0.975]$	-
k_A	Relative size of animal reservoir population	$\Gamma(1.26, 19.3)$	$[1.18, 18.3, 81.4]$	-

*Where Exp(.), $\Gamma(.)$ and B(.) are the exponential, gamma (parameterised with shape and scale) and beta distributions, respectively.

†Former province-specific priors used for η_H^{post} and γ_H^{post}; prior distributions and percentiles for Bandundu presented, see Table D for other former provinces.

¶The combined treatment and disease-induced death rate from stage 2, before 1998, was: $\gamma_H^{\text{pre}} = b_{\gamma_H^{\text{pre}}} \gamma_H^{\text{post}}$.

‡d_{change} is only fitted in the former province of Bandundu.

§$\eta_{H_{\text{amp}}}$, $\gamma_{H_{\text{amp}}}$ and d_{steep} are only fitted in the former provinces of Bandundu and Bas Congo; the prior distributions and percentiles presented here relate to Bandundu, see Table D for Bas Congo.

S1.3 Modelling passive detection and its improvement

Our new model fitting, for the model with animal transmission, follows the same methods for modelling passive detection and its improvement as Crump et al. [7] and this is described below for completeness.

There are two sources of passive detection improvements considered in our model: a rapid improvement due to the introduction of the card agglutination test for trypanosomes (CATT) test in all health zones in 1998 and a gradual improvement over time in the former Bandundu and Bas Congo provinces around 2008 and mid-2015 respectively. Prior distributions and percentiles of parameters related to passive detection and its improvement over time are summarised in Table D.

For improvements from 1998 we use the following equations to describe transmission rates from infected classes:

$$\eta_H(Y) = \eta_H^{\text{post}} \left[1 + \frac{\eta_{H_{\text{amp}}}}{1 + \exp\left(-d_{\text{steep}}(Y - d_{\text{change}})\right)} \right] \tag{S1.3.1}$$

$$\gamma_H(Y) = \gamma_H^{\text{post}} \left[1 + \frac{\gamma_{H_{\text{amp}}}}{1 + \exp\left(-d_{\text{steep}}(Y - d_{\text{change}})\right)} \right] \tag{S1.3.2}$$

We assume that all stage 1 cases are reported, but that some of the exits from stage 2 are due to death from gHAT disease. In 1998 the reporting probability for an exit from stage 2 is given by u, however as the exit rate from stage 2 increases this reporting probability does not stay constant, but increases (proportionally more people would be detected and treated with higher exit rates). When we compute reporting rates from stage 2 we therefore use the following:

$$\text{Death rate} = (1-u)\gamma_H^{\text{post}} \tag{S1.3.3}$$

$$\text{Stage 2 reporting incidence} = (\gamma_H(Y) - \text{Death rate})(I_{2H1} + I_{2H4}) \tag{S1.3.4}$$

Improvement in passive screening systems over time is considered across the whole of Bandundu and Bas Congo. The province level staging data in Bandundu suggested that passive surveillance systems in Bandundu have improved over time, which was confirmed by PNLTHA and is also supported by previous modelling work [1]. In Bas Congo, FIND implemented the use of rapid diagnostic tests from 2015. Staging information which was available at the province-level from 2000–2012 from the paper of Lumbala et al. [11], and in the HAT Atlas data for 2015 and 2016. To inform the health-zone-level analyses, a province-level fit was carried out to the staged case data of Lumbala et al. augmented with the HAT Atlas data aggregated to the former province level for the years 2013–2016. These analyses provided no evidence for improvement in passive screening systems (in line with the simple sigmoidal model assumed) for any provinces other than Bandundu and Bas Congo. For Bandundu and Bas Congo, gamma distributions were fitted to the province level posterior samples of $\eta_{H_{\text{amp}}}$, $\gamma_{H_{\text{amp}}}$ and d_{steep}. The shape (k) and scale (θ) for these fitted distributions were used as the parameters of gamma prior distributions of $\eta_{H_{\text{amp}}}$, $\gamma_{H_{\text{amp}}}$ and d_{steep} in all health zones of Bandundu and Bas Congo. In Bandundu health zones a scaled and shifted beta distribution was used as the prior for d_{change}. The use of a broader prior for d_{change} than would have resulted from using the province–level posterior distribution resulted from comparing aggregate health-zone-level results with province-level observed data. In Bas Congo health zone a fixed value of 2015.5 was used for d_{change}.

Priors for the health-zone-level η_H^{post} and γ_H^{post} parameters were also informed by the province-level fits. Gamma prior distributions were used which had the same mode $(\text{mode} = (k-1)\,\theta)$ as Gamma distributions fitted to the province-level posterior distribution of the parameters; and a standard deviation $(s.d. = \sqrt{k}\theta)$ of 3×10^{-3} for η_H^{post} and 1×10^{-4} for γ_H^{post}, being arbitrarily selected higher variation than the province-level posterior distributions.

Table D: **Parameterisation of passive detection improvement.** Notation and brief description of fitted parameters related to passive detection improvement plus their within former province prior distributions and [2.5th, 50th & 97.5th] percentile.

Parameter			
	**Province**	**Prior distribution**	**Percentiles of prior distribution**
η_H^{post} – Treatment rate from stage 1, 1998 onwards			
	**Bandundu**	$\Gamma(3.54, 5.32 \times 10^{-5})$	$[4.59, 17.1, 42.9] \times 10^{-5}$
	**Bas Congo**	$\Gamma(12.0, 2.89 \times 10^{-5})$	$[1.78, 3.36, 5.68] \times 10^{-4}$
	**Equateur**	$\Gamma(4.92, 4.51 \times 10^{-5})$	$[7.12, 20.7, 45.7] \times 10^{-5}$
	**Kasai Occidental**	$\Gamma(10.9, 3.03 \times 10^{-5})$	$[1.64, 3.20, 5.53] \times 10^{-4}$
	**Kasai Oriental**	$\Gamma(2.90, 5.87 \times 10^{-5})$	$[3.38, 15.1, 41.5] \times 10^{-5}$
	**Katanga**	$\Gamma(1.29, 8.79 \times 10^{-5})$	$[5.88, 86.2, 376] \times 10^{-6}$
	**Kinshasa**	$\Gamma(1.26, 8.91 \times 10^{-5})$	$[5.44, 84.4, 376] \times 10^{-6}$
	**Maniema**	$\Gamma(4.25, 4.85 \times 10^{-5})$	$[5.90, 19.0, 44.3] \times 10^{-5}$
	**Orientale**	$\Gamma(1.16, 9.27 \times 10^{-5})$	$[4.24, 79.0, 373] \times 10^{-6}$
γ_H^{post} – Treatment rate from stage 2, 1998 onwards			
	**Bandundu**	$\Gamma(2.45, 1.92 \times 10^{-3})$	$[7.59, 40.7, 121] \times 10^{-4}$
	**Bas Congo**	$\Gamma(1.48, 2.47 \times 10^{-3})$	$[2.54, 28.7, 114] \times 10^{-4}$
	**Equateur**	$\Gamma(1.95, 2.15 \times 10^{-3})$	$[4.88, 35.0, 118] \times 10^{-4}$
	**Kasai Occidental**	$\Gamma(1.71, 2.29 \times 10^{-3})$	$[3.65, 31.9, 116] \times 10^{-4}$
	**Kasai Oriental**	$\Gamma(1.49, 2.46 \times 10^{-3})$	$[2.60, 28.8, 114] \times 10^{-4}$
	**Katanga**	$\Gamma(1.53, 2.43 \times 10^{-3})$	$[2.78, 29.4, 115] \times 10^{-4}$
	**Kinshasa**	$\Gamma(1.68, 2.31 \times 10^{-3})$	$[3.50, 31.5, 116] \times 10^{-4}$
	**Maniema**	$\Gamma(2.60, 1.86 \times 10^{-3})$	$[8.45, 42.3, 122] \times 10^{-4}$
	**Orientale**	$\Gamma(2.54, 1.88 \times 10^{-3})$	$[8.11, 41.7, 122] \times 10^{-4}$
$\eta_{H_{\text{amp}}}$ – Relative improvement in passive stage 1 detection rate			
	**Bandundu**	$\Gamma(2.01, 1.05)$	$[0.258, 1.77, 5.87]$
	**Bas Congo**	$\Gamma(5.23, 1.70)$	$[2.98, 8.33, 18.0]$
$\gamma_{H_{\text{amp}}}$ – Relative improvement in passive stage 2 detection rate			
	**Bandundu**	$\Gamma(1.001, 5)$	$[0.127, 3.47, 18.5]$
	**Bas Congo**	$\Gamma(1.46, 1.26)$	$[0.126, 1.45, 5.81]$
d_{steep} – Speed of improvement in passive detection rate			
	**Bandundu**	$\Gamma(39.6, 2.70 \times 10^{-2})$	$[0.761, 1.06, 1.42]$
	**Bas Congo**	$\Gamma(3.21, 1.45)$	$[1.03, 4.18, 10.9]$

S1.4 Modelling vector control

In the present study we utilise the same method to simulate the impact of biannual vector control on tsetse populations as presented elsewhere [17, 7, 10]. We model the dynamics of tsetse populations in the presence of Tiny Target-based vector control on the basis of an assumed annual tsetse population density reduction. The assumed annual reductions are based on reported percentage reductions in tsetse population density in the first year of Tiny Target deployments [6, 21, 22]. Fig A shows the assumed dynamics of tsetse populations where targets are either moderately effective, with a 60% annual tsetse population density reduction (lower than observed in Guinea in the first year of deployment [6]), or highly effective, with a 90% tsetse population density reduction per year (as reported in Uganda [21] and Yasa Bonga health zone in the DRC [22] following the first year of deployments). In this analysis we used the 90% annual tsetse population density reduction [22] formulation in Yasa Bonga health zone as part of fitting, but 60% elsewhere for the main text results as a low, conservative estimate of what large-scale Tiny Target deployments might achieve. Results using more optimistic annual tsetse population density reductions of 80% and 90% are available online through our graphical user interface (GUI; `https://hatmepp.warwick.ac.uk/animalfitting/v1/`).

The function which describes the probability of a host-seeking tsetse both hitting a Tiny Target and dying as a result, f_T, is time dependent (t, in days) from when the targets were first deployed:

$$f_T(t) = f_{\text{max}} \left(1 - \frac{1}{1 + \exp(-0.068(\text{mod}(t, 182.5) - 127.75))}\right) \tag{S1.4.1}$$

and f_{max} - the maximum daily probability of contacting a Tiny Target and dying as a result. f_T modifies all the bite rates α in our tsetse equations to produce an additional Tiny-Target-induced mortality for tsetse. f_{max} is chosen such that the tsetse population after one year is at the observed/assumed percentage reduction. For this model of f_T this is given by $f_{\text{max}} = 0.0305$ and $f_{\text{max}} = 0.0750$ for annual tsetse population density reductions of 60% and 90%, respectively. The value 182.5 reflects twice-yearly deployments of Tiny Targets, as used in the DRC [22]. The effectiveness of Tiny Targets is assumed to wane over time so that at the point of the next deployment, the previous deployment is virtually non-effective. To simulate this we have a rapid decrease in effectiveness after 127.75 days, determined by the value -0.068, after this time [17]. This represents lost Tiny Targets (e.g. due to rainfall) or loss of the effectiveness of remaining targets (e.g. vegetation growth impacting their visibility).

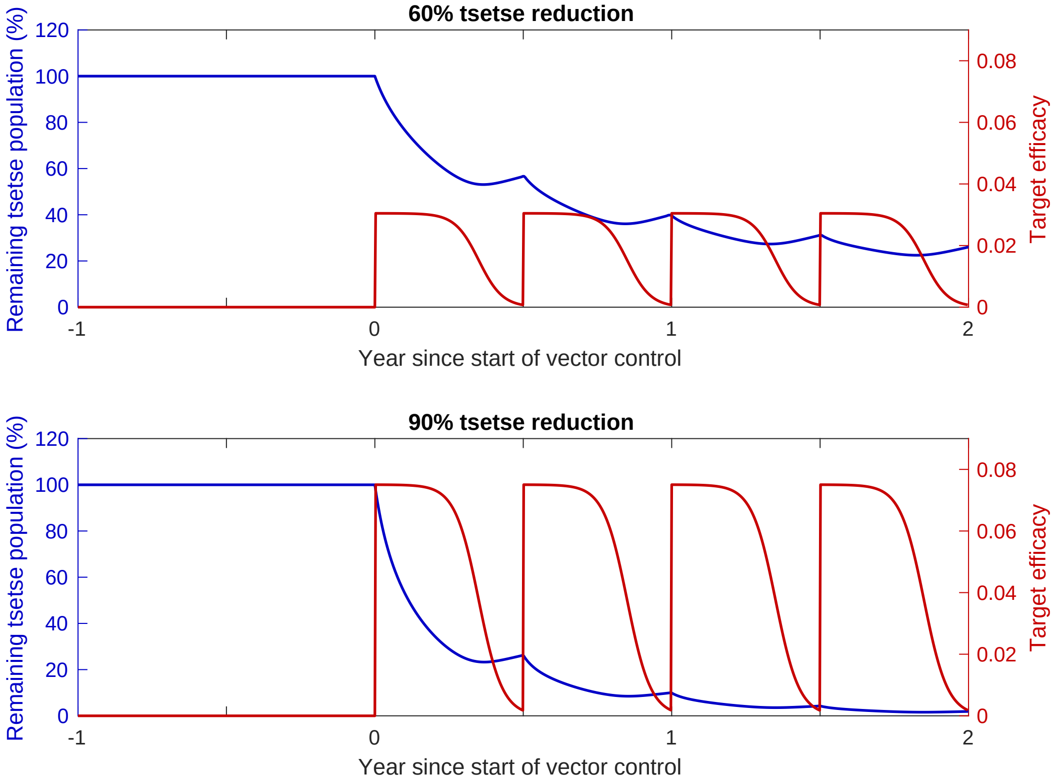

Fig A: **Impact of Tiny Targets on tsetse density.** The figures show how varying target efficacy (red line) impacts tsetse population density (blue line). Target efficacy is measured as the proportion of a host-seeking tsetse which will both hit the Tiny Target and die as a result. The graphs, reproduced from [17] under a CC-BY licence, show the necessary efficacy of targets needed to reduce density by 60% (top) and 90% (bottom) by the end of the first year.

S1.5 Fitting

S1.5.1 Running the models

Our ODE models were run assuming that, prior to 1998, they were at their endemic equilibrium. This endemic equilibrium was calculated analytically, based on model parameterisation, and used as an initial condition in the model code. In 1998 we assume active screening began at the same level reported in 2000 and that there was improvement to passive screening due to the availability of the CATT diagnostic test. In our model this has the effect of perturbing the dynamics away from endemic equilibrium and reducing transmission.

For fitting the models, there are two elements, each of which is initialised.

- Two chains are run in the Markov chain Monte Carlo (MCMC) used in the fitting. The chains are initialised using the fixed parameters and by random perturbations around supplied, individually valid, initial values of each parameter being fitted, rejecting those parameter sets that do not produce a valid posterior probability.
- Each projection is initialised with a randomly sampled realisation from the posterior distribution of fitted parameters alongside the set of fixed parameters.

S1.5.2 Likelihood

Eight parameters; $R_0, r, \eta_H, \gamma_H, b_{\gamma_{H0}}, k_1, u$, and Spec were fitted in all health zones for both models. A further two parameters; k_A and f_A, were fitted in all health zones for the model with animal transmission. Additional parameters were included as required (combinations of d_{change}, $\eta_{H_{\text{amp}}}$, $\gamma_{H_{\text{amp}}}$, d_{steep} and $b_{\text{specificity}}$ as appropriate, see above).

For fitting the model to case data we transform model ODE solutions (for S1.2.1) into annual case reporting denoted A_{M1}, A_{M2}, for active stage 1 and stage 2 and P_{M1}, P_{M2}, for passive stage 1 and 2. Since we always know the stage (1 or 2) in the model simulations there is no requirement for a "U" (unknown stage) category for the model. These are computed using solutions to the ODEs for the given set of parameters aggregated across a year.

In terms of main text Fig 1, they relate to the transfer from infectious categories to the recovered category – the new annual reported case incidence. This is either by passive detection from stage 1 for year Y:

$$P_{M1}(Y) = \int_Y^{Y+1} \eta_H(Y)\big(I_{1H1}(t) + I_{1H4}(t)\big)\,\mathrm{d}t,$$

passive detection from stage 2

$$P_{M2}(Y) = \int_Y^{Y+1} (\gamma_H(Y) - \text{Death rate})\big((I_{2H1}(t) + I_{2H4}(t)\big)\,\mathrm{d}t,$$

or by active screening from the low-risk $(H1)$ group in year Y

$$A_{M1}(Y) = z(Y)\text{Sens}I_{1H1}(Y) + z(Y)(1 - \text{Spec})\big(k_1 N_H - I_{1H1}(Y) - I_{2H1}(Y)\big)$$

and

$$A_{M2}(Y) = z(Y) \times \text{Sens} \times I_{2H1}(Y)$$

with variable active screening coverage by year, $z(Y)$ and fixed diagnostic sensitivity. A_{M1} also contains any false positives that may have been incorrectly identified from non-infected people based on the high but imperfect specificity of the active screening algorithm. We assume in the DRC that all false positives would be assigned to be stage 1 and treated, however in the model false positives stay in the susceptible class unlike true cases which move to recovereds.

The log-likelihood function used in the adaptive Metropolis-Hastings MCMC contained two terms in each year for which reported case numbers were available for each source of reported cases (active or passive screening). These were:

- a beta-binomial probability that the total number of cases reported in that year for that source came from the available population (either the reported number of people actively screened for active screening, or the health zone population for passive screening) with probability calculated from solving the ODE for the current set of parameters, and

- a binomial probability that the reported stage 1 cases come from the total number of reported staged cases where the probability parameter again comes from the solution of the ODE. In many years staging is unknown and so this part of the log-likelihood will return zero and not contribute to our calculation. In some years, we only partially know staging information.

This formulation allowed over-dispersion in the observed cases to be included, via the beta-binomial distribution, and any proportion of cases with reported disease stage to be appropriately accounted for (assuming that the reporting of staging information is independent of the disease stage). The log-likelihood function was as follows:

$$
\begin{aligned}
LL(\theta|x) = & \log(P(x|\theta)) \\
\propto & \sum_{i=2000}^{2016} \Bigg(\log\left[\mathrm{BetaBin}\left(A_{D1}(i) + A_{D2}(i) + A_{DU}(i); z(i), \frac{A_{M1}(i) + A_{M2}(i)}{z(i)}, \mathrm{disp}_{\mathrm{act}}\right)\right] \\
& + \log\left[\mathrm{Bin}\left(A_{D1}(i); A_{D1}(i) + A_{D2}(i), \frac{A_{M1}(i)}{A_{M1}(i) + A_{M2}(i)}\right)\right] \\
& + \log\left[\mathrm{BetaBin}\left(P_{D1}(i) + P_{D2}(i) + P_{DU}(i); N_H, \frac{P_{M1}(i) + P_{M2}(i)}{N_H}, \mathrm{disp}_{\mathrm{pass}}\right)\right] \\
& + \log\left[\mathrm{Bin}\left(P_{D1}(i); P_{D1}(i) + P_{D2}(i), \frac{P_{M1}(i)}{P_{M1}(i) + P_{M2}(i)}\right)\right]\Bigg)
\end{aligned}
$$

The model takes parameterisation θ, x is the data, $P_{Dj}(i)$ and $A_{Dj}(i)$ are the number of cases detected by passive or active screening (of stage j, which may be 1, 2 or unknown, U) in year i of the data. $P_{Mj}(i)$ and $A_{Mj}(i)$ are the number of cases detected by passive or active screening (of stage j) in year i of the model, and $z(i)$ is the number of people actively screened in year i. $\mathrm{BetaBin}(m; n, p, \rho)$ gives the probability of obtaining m successes out of n trials with probability p and overdispersion parameter ρ. The overdispersion accounts for larger variance than under the binomial. The probability density function of this distribution is given by:

$$
\mathrm{BetaBin}(m; n, p, \rho) = \frac{\Gamma(n+1)\Gamma(m+a)\Gamma(n-m+b)\Gamma(a+b)}{\Gamma(n-m+1)\Gamma(n+a+b)\Gamma(a)\Gamma(b)}
$$

where $a = p(1/\rho - 1)$ and $b = a(1-p)/p$.

S1.5.3 Imputation of missing numbers screened information

There are instances in the data where the number of cases from active screening within year t ($A_D(t) = A_{D1} + A_{D2}$) is not consistent with the number of people recorded as having been screened in that year for that health zone ($z(t)$), i.e. $A_D(t) > z(t)$. In this situation, if $A_D(t) < 20$ we assume that these people have attended a screening outside their home health zone and the record has been correctly allocated to their home health zone and we set $z(t) = A_D(t)$. However, where $A_D(t) \geq 20$ we assume that a screening must have taken place in the health zone but that only the positive test results have been recorded. To allow use of these records we impute the missing negative test results ($A_D^-(t)$) during the MCMC analysis, and this enables the model to account for perturbation of the system by unknown high levels of active screening activity within a year.

We use a prior conditional on the other model parameters (θ) for $A_D^-(t)$; $A_D^-(t)|\theta \sim \mathrm{NB}(A_D(t), P^+(\theta))$, where $P^+(\theta)$ is the probability of a positive active screening result given the current θ (obtained from solutions of the ODE up to this point in time). The proposal distribution for $A_D^-(t)$ is the same as the conditional prior. The value $\hat{z}(t) = A_{D1} + A_{D2} + A_D^-(t)$ is used in place of the unknown $z(t)$ in the likelihood calculation.

Table E: **Imputation of negative active screening results.** Former province, health zone, year and case numbers from active screening $(A_D(t))$ where imputation of $A_D^-(t)$ was performed; plus median and 95% credible intervals of posterior distributions of $A_D^-(t)$.

Former province	Health zone	Year (t)	$A_D(t)$	$A_D^-(t)$	
				Median	[95% CI]
Kinshasa	Maluku 2	2000	30	6052	[4020, 9101]
		2001	102	19653	[13792, 28992]
	Mont Ngafula 1	2000	303	214019	[125975, 277536]
	Nsele	2001	147	98902	[70301, 149644]
Orientale	Doruma	2007	320	17996	[15550, 20466]
		2008	152	13668	[10906, 16676]
		2011	215	40324	[29970, 48452]

S1.6 Host-specific contributions to transmission

For the model with animals contributing to transmission of gHAT, the basic reproduction number specific to animals and humans can be calculated. This process is carried out for each of 2 000 samples from the joint posterior distribution of the parameters resulting from an MCMC analysis performed as in [7].

We construct a next generation matrix $\mathbf{K} = -\mathbf{T}\mathbf{\Sigma}^{-1}$, where $\mathbf{T}$ and $\mathbf{\Sigma}$ are 12×12 transmission and transition matrices with elements $t_{i,j}$ or $s_{i,j}$, respectively. The two dimensions of $\mathbf{T}$ and $\mathbf{\Sigma}$ map to compartments of the model thus: E_{H1}, I_{1H1}, I_{2H1}, E_{H4}, I_{1H4}, I_{2H4}, E_A, I_A, E_{1V}, E_{2V}, E_{3V}, and finally I_V, see the main text Fig 1. That is, three compartments relating to low-risk humans, three compartments relating to high-risk humans, two compartments relating to reservoir animals and four compartments relating to tsetse vectors. As R_0 is defined in the absence of new intervention measures, active screening, vector control, nor improvements to passive detection rates are included. Equations for the non-zero elements of T and Σ are presented in Tables F and G, respectively.

The overall system reproduction number, R_0, is the spectral radius (dominant eigenvalue) of $\mathbf{K}$ denoted by $\rho(\mathbf{K})$. Similarly, to compute the human-specific or animal-specific reproduction numbers, R_{0_H} and R_{0_A}, we use

$$R_{0_\mathrm{i}} = \rho((\mathbf{P}_v + \mathbf{P}_i)\mathbf{K}), \qquad i \in \{H, A\}$$

with

$$\mathbf{P}_v = \begin{bmatrix} \mathbf{0}_{8,8} & \mathbf{0}_{8,4} \\ \mathbf{0}_{4,8} & \mathbf{I}_4 \end{bmatrix}$$

$$\mathbf{P}_H = \begin{bmatrix} \mathbf{I}_6 & \mathbf{0}_{6,6} \\ \mathbf{0}_{6,6} & \mathbf{0}_{6,6} \end{bmatrix}$$

$$\mathbf{P}_A = \begin{bmatrix} \mathbf{0}_{6,6} & \mathbf{0}_{6,2} & \mathbf{0}_{6,4} \\ \mathbf{0}_{2,6} & \mathbf{I}_2 & \mathbf{0}_{2,4} \\ \mathbf{0}_{4,6} & \mathbf{0}_{4,2} & \mathbf{0}_{4,4} \end{bmatrix}$$

If both $R_{0_\mathrm{H}} < 1$ and $R_{0_\mathrm{A}} < 1$, neither host group is classified as a maintenance reservoir, but both are necessary for disease persistence. If $R_{0_\mathrm{H}} > 1$ and $R_{0_\mathrm{A}} < 1$, humans form a maintenance reservoir (can sustain disease without transmission in animals) and are necessary to have endemic disease.

Table F: **Next generation matrix; non-zero elements of the transmission matrix, T.** Equations for $t_{i,j}$ under the model with animal transmission.

Transmission	Element, $t_{i,j}$	Value
I_V infects E_{H1}	$t_{1,12}$	$\lambda_{H1} = \alpha m_{\mathrm{eff}} f_H \frac{k_1}{k_1+rk_4}$
I_V infects E_{H4}	$t_{4,12}$	$\lambda_{H4} = \alpha m_{\mathrm{eff}} f_H \frac{rk_4}{k_1+rk_4}$
I_V infects E_A	$t_{7,12}$	$\lambda_A = \alpha m_{\mathrm{eff}} f_A$
I_{1H1} infects E_{1V}	$t_{9,2}$	$\alpha f_H \frac{k_1}{k_1+rk_4} p_V \frac{(S_V^*+\varepsilon G_V^*)}{k_1 N_H}$
I_{2H1} infects E_{1V}	$t_{9,3}$	$= t_{9,2}$
I_{1H4} infects E_{1V}	$t_{9,5}$	$\alpha f_H \frac{rk_4}{k_1+rk_4} p_V \frac{(S_V^*+\varepsilon G_V^*)}{k_4 N_H}$
I_{2H4} infects E_{1V}	$t_{9,6}$	$= t_{9,5}$
I_A infects E_{1V}	$t_{9,8}$	$\alpha f_A p_V \frac{(S_V^*+\varepsilon G_V^*)}{k_A N_H}$

See main text Fig 1 for model compartment information, Tables B and C for definitions of most parameters, and:

m_{eff} is the tsetse-to-human relative density (from R_0);

$S_V^* = \frac{\mu_V N_H}{\alpha+\mu_V}$ is the disease-free equilibrium value of S_V; and

$G_V^* = \frac{\alpha N_H}{\alpha+\mu_V}$ is the disease-free equilibrium value of G_V.

Table G: **Next generation matrix; non-zero elements of the transition matrix, $\boldsymbol{\Sigma}$.** Equations for $s_{i,j}$ under the model with animal transmission.

Transition	Element, $s_{i,j}$	Value
Leaves E_{H1}	$s_{1,1}$	$-\sigma_H - \mu_H$
Enters I_{1H1} from E_{H1}	$s_{2,1}$	σ_H
Leaves I_{1H1}	$s_{2,2}$	$-\eta_H^{\text{pre}} - \phi_H - \mu_H$
Enters I_{2H1} from I_{1H1}	$s_{3,2}$	ϕ_H
Leaves I_{2H1}	$s_{3,3}$	$-\gamma_H^{\text{pre}} - \mu_H$
Leaves E_{H4}	$s_{4,4}$	$-\sigma_H - \mu_H$
Enters I_{1H4} from E_{H4}	$s_{5,4}$	σ_H
Leaves I_{1H4}	$s_{5,5}$	$-\eta_H^{\text{pre}} - \phi_H - \mu_H$
Enters I_{2H4} from I_{1H4}	$s_{6,5}$	ϕ_H
Leaves I_{2H4}	$s_{6,6}$	$-\gamma_H^{\text{pre}} - \mu_H$
Leaves E_A	$s_{7,7}$	$-\sigma_A - \mu_A$
Enters I_A from E_A	$s_{8,7}$	σ_A
Leaves I_A	$s_{8,8}$	$-\mu_A$
Leaves E_{1V}	$s_{9,9}$	$-3\sigma_V - \mu_V$
Enters E_{2V} from E_{1V}	$s_{10,9}$	$3\sigma_V$
Leaves E_{2V}	$s_{10,10}$	$-3\sigma_V - \mu_V$
Enters E_{3V} from E_{2V}	$s_{11,10}$	$3\sigma_V$
Leaves E_{3V}	$s_{11,11}$	$-3\sigma_V - \mu_V$
Enters I_V from E_{3V}	$s_{12,11}$	$3\sigma_V$
Leaves I_V	$s_{12,12}$	$-\mu_V$

See main text Fig 1 for model compartment information, Tables B and C for model parameter definitions.

S1.7 Model comparison

We compare our models on the basis of Bayes factors; $\mathrm{BF}_i = \pi(\mathbf{x}|i) / \pi(\mathbf{x}|j)$, where $\pi(\mathbf{x}|m)$ is the marginal likelihood, or *evidence*, of the data $\mathbf{x}$ for model m. Here, $m \in \{w, wo\}$ indicates either the Warwick gHAT model variant with (w) or without (wo) animals contributing to transmission (Models 7 and 4 respectively in previous publications [16, 12, 18]), depending on whether we are considering the statistical support for the model with or without animal transmission relative to the other model being studied. The use of Bayesian model evidence makes use of the full probability distribution of the model rather than a point estimate (usually the maximum likelihood estimate) and naturally accounts for differences in the number of parameters required for different models.

An importance sampled estimator of the model evidence was implemented following Touloupou et al. [23].

The joint distribution of $(\boldsymbol{\theta}_m, \mathbf{x})$, for parameters $\boldsymbol{\theta}_m = (\theta_1, \theta_2, \ldots, \theta_{d_m})$ of model m and data $\mathbf{x} = (x_1, x_2, \ldots, x_n)$ satisfies

$$\pi(\boldsymbol{\theta}_m|\mathbf{x})\,\pi(\mathbf{x}|\mathbf{m}) = \pi(\mathbf{x}|\boldsymbol{\theta}_m)\,\pi(\boldsymbol{\theta}_m)\,, \tag{S1.7.1}$$

where $\pi(\boldsymbol{\theta}_m|\mathbf{x})$ is the joint posterior distribution of parameters $1 \ldots d$, $\pi(\mathbf{x}|\mathbf{m})$ is the marginal likelihood or *evidence*; $\pi(\mathbf{x}|\boldsymbol{\theta}_m)$ is the likelihood, and $\pi(\boldsymbol{\theta}_m)$ is the prior distribution.

By use of MCMC methods to investigate the posterior distribution of the parameters, calculation of $\pi(\mathbf{x}|\mathbf{m})$ is avoided. Calculation of the evidence for use in model comparison requires computing the integral:

$$\begin{aligned}\pi(\mathbf{x}|\mathbf{m}) &= \int \pi(\mathbf{x}|\boldsymbol{\theta}_m)\,\pi(\boldsymbol{\theta}_m)\;\mathrm{d}\boldsymbol{\theta}_m & \text{(S1.7.2)}\\ &= \int \pi(\mathbf{x}|\boldsymbol{\theta}_m)\,\frac{\pi(\boldsymbol{\theta}_m)}{q(\boldsymbol{\theta}_m)}q(\boldsymbol{\theta}_m)\;\mathrm{d}\boldsymbol{\theta}_m & \text{(S1.7.3)}\end{aligned}$$

Equation S1.7.2 cannot be calculated analytically except for some small set of tractable models. It can, however, be rewritten as equation S1.7.3, where $q(\boldsymbol{\theta}_m)$ is a d_m-dimensional probability density function. From this, an importance sampled estimator of $\pi(\mathbf{x}|\mathbf{m})$ is:

$$\hat{P}_q = \frac{1}{N}\sum_{i=1}^{N}\pi(\mathbf{x}|\boldsymbol{\theta}_{m,i})\,\frac{\pi(\boldsymbol{\theta}_{m,i})}{q(\boldsymbol{\theta}_{m,i})}, \tag{S1.7.4}$$

where the $\boldsymbol{\theta}_{m,i}$ are N samples drawn from q.

A defence mixture [9] was used for $q(\boldsymbol{\theta}_m)$:

$$q(\boldsymbol{\theta}_m) = p\phi(\boldsymbol{\theta}^*_m; n, \boldsymbol{\mu}_1 \ldots \boldsymbol{\mu}_n, \mathbf{C}_1 \ldots \mathbf{C}_n)\left|\frac{\boldsymbol{\theta}^*_m}{\boldsymbol{\theta}_m}\right| + (1-p)\,\pi(\boldsymbol{\theta}_m) \tag{S1.7.5}$$

where $\phi(\cdot)$ is a mixture of n multivariate Gaussian distributions with means $\boldsymbol{\mu}_j$ ($j = \{1 \ldots n\}$), and covariance matrices $\mathbf{C}_j$, $\left|\frac{\boldsymbol{\theta}^*_m}{\boldsymbol{\theta}_m}\right|$ is the Jacobian transformation relating probability on transformed and original scales, and p is a mixing proportion ($p = 0.95$ was chosen for use, being a typical value [23]).

In each of our health-zone-level MCMC analyses of the models with and without animal transmission, 2 000 samples from the joint posterior distribution were generated and stored, and $\phi(\boldsymbol{\theta}_m; n, \boldsymbol{\mu}_1 \ldots \boldsymbol{\mu}_n, \mathbf{C}_1 \ldots \mathbf{C}_n)$ for each health zone and model was chosen using the Matlab `fitgmdist` function, selecting n based on Akaike's Information Criterion (AIC). To account for the high correlations between some of our model parameters, regularisation was applied to ensure that the covariance matrices, $\mathbf{C}_k$, would be positive semi-definite. Before passing to `fitgmdist`, transformations were applied to the posterior samples to put them in the range $(-\infty, \infty)$ – appropriate for Gaussian distributions – followed by scaling and centring to keep the regularisation consistent across analyses, at least at the simple, single overall covariance matrix level.

Having defined $\phi(\cdot)$ for a given analysis (health zone, model combination), $\hat{P}_q$ was calculated (equation S1.7.4) using $N = 2\,000$ samples drawn from $q(\boldsymbol{\theta}_m)$.

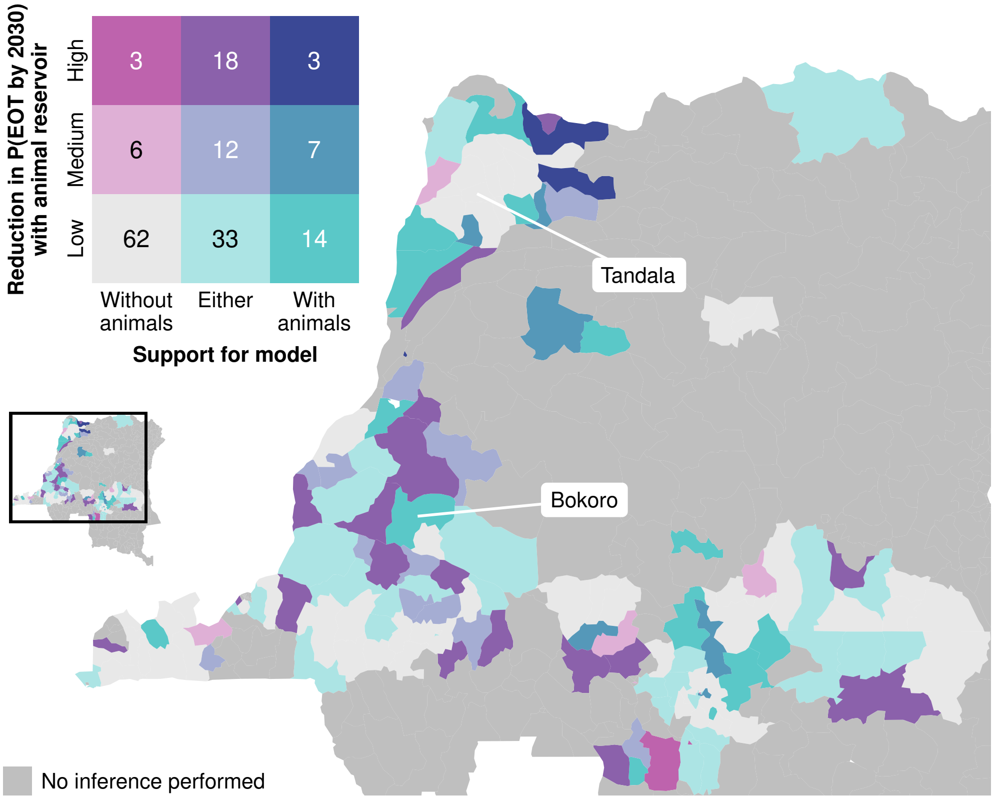

Fig B: Bivariate choropleth showing support for the models with or without animals contributing to transmission and the difference in the probability (P_d) of achieving EoT to humans by 2030 from these two models ("High" is more than 10% difference, "Medium" is 5-10% difference, and "Low" is less than 5% difference). Shapefiles used to produce these map were provided by Nicole Hoff and Cyrus Sinai under a CC-BY licence (current versions can be found at `https://data.humdata.org/dataset/drc-health-data`).

S1.8 Additional results

Fig B is a bivariate choropleth map which combines support for models with and without animal transmission with difference in the probability of achieving end of transmission by 2030 under the strategy where active screening in the health zone continues at the mean level observed in the period 2012–2016 ($P_d = \mathbb{P}(\text{EOT by 2030}|\text{Model without animal transmission}) - \mathbb{P}(\text{EOT by 2030}|\text{Model with animal transmission})$. For presentation in Fig B both variables were put into three categories: without animals $(\mathrm{BF_{wo}} > 10^{\frac{1}{2}})$, either $(\mathrm{BF_{wo}} < 10^{\frac{1}{2}} \wedge \mathrm{BF_w} < 10^{\frac{1}{2}})$, and with animals $(\mathrm{BF_w} > 10^{\frac{1}{2}})$ for model support; and for difference in probability of achieving EoT by 2030 (P_d): low $(P_d \leq 0.05)$, medium $(0.05 < P_d \leq 0.1)$, and high $(P_d > 0.1)$.

There are 18 health zones (dark purple) with (i) more than 10% reduction in probability of meeting the EoT goal under the model with animal transmission compared to the model without animal transmission and (ii) with weak support for either model variant. In these locations there is considerable uncertainty in whether animals contribute to transmission and this could alter policy recommendations for future strategy based on model predictions. In three health zones (dark blue) there is strong evidence for animal transmission and more than 10% reduction in probability of EoT by 2030 and so these health zones (all in former Equateur province) have more pessimistic predictions than indicated in previous modelling work [10].

S1.8.1 Ensemble model

Model projections were carried out taking 1 000 random samples from the joint posterior distribution of the model parameters, using these to simulate the deterministic model and from each of these generating 10 observational samples for case reporting using the beta-binomial distributions described earlier. The end of transmission was computed using the deterministic ODE outputs for new human infections by applying a proxy threshold of less than one new infection within a year; stochastic samples are not applied to this transmission process. As a result, there were 1 000 EoT estimates and 10 000 case reporting samples for each of the models

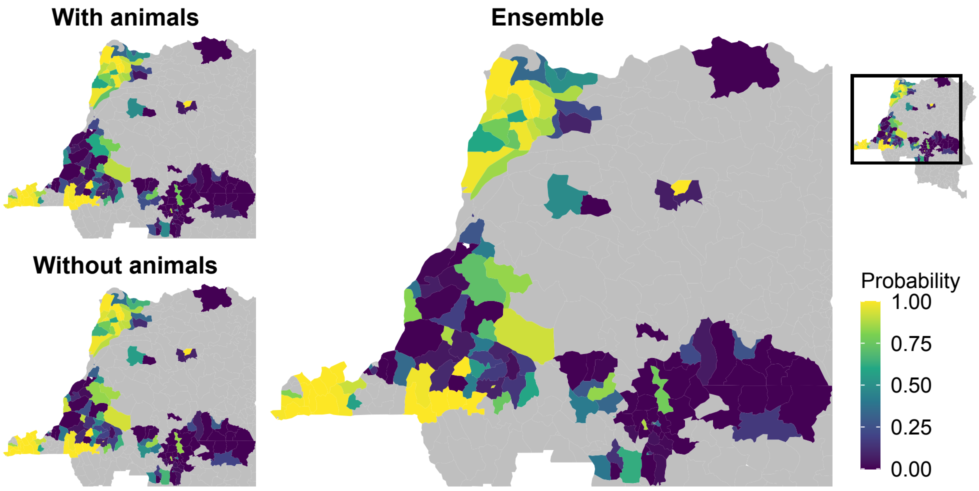

Fig C: Maps of the probability that end of transmission in humans is achieved by 2030 for the models with and without animal transmission, and an ensemble model of these. Active screening post-2016 was assumed to be at the mean level observed 2012–2016, passive screening continued at the 2016 level of effectiveness, and no vector control was performed (except in Yasa Bonga health zone). Shapefiles used to produce these map were provided by Nicole Hoff and Cyrus Sinai under a CC-BY licence (current versions can be found at `https://data.humdata.org/dataset/drc-health-data`).

(with and without animal transmission) relating to the end of transmission. Bayesian model averaging was used to produce an ensemble model to predict the probability of EoT by randomly sampling posterior N_{wo} samples from the model without animal transmission, and $1000 - N_{\text{wo}}$ posterior samples taken from the model with animal transmission, where $N_{\text{wo}} \sim \text{Bin}\left(1000, \frac{\text{BF}_{\text{wo}}}{\text{BF}_{\text{wo}}+1}\right)$.

Fig C shows the probability of achieving EoT by 2030 in each health zone, for the ensemble model and the individual models with and without animal transmission. As a result of the scale covering the full range of probability from 0 to 1 to reflect the between health zone variation in probability and the between model, within health zone differences being far smaller than this, there is little visually discernible difference between the presented maps of probability, with the ensemble model being a within health zone weighted mixture of the outcomes from the other two.

The percentage of the studied health zones expected to reach EoT by the each of the years 2017–2040 is given in Fig D. This is equivalent to Fig 8 in the main text, with the addition of results from the ensemble model.

S1.8.2 Prevalence of infection

For the model with animal transmission, the prevalence of infection was calculated for 1 000 samples from the posterior distribution of fitted parameters. The within-year mean of the number of stage 1 and 2 in low- and high-risk population groups (groups 1 and 4, respectively) (I_{1H1}, I_{2H1}, I_{1H4} and I_{2H4}) was used for the human prevalence, and the within-year mean of I_A for the animal prevalence. For vectors, we assume that infections may be detected anywhere within the tsetse (e.g. by molecular screening of the sampled tsetse which could include mid-gut infections), or specifically in the salivary glands (e.g. by dissection and microscopy) from where the infection can be passed on to host species. Therefore, both all infection and salivary gland infection prevalences were calculated, using the within-year mean of $E_{1V} + E_{2V} + E_{3V} + I_V$ or I_V.

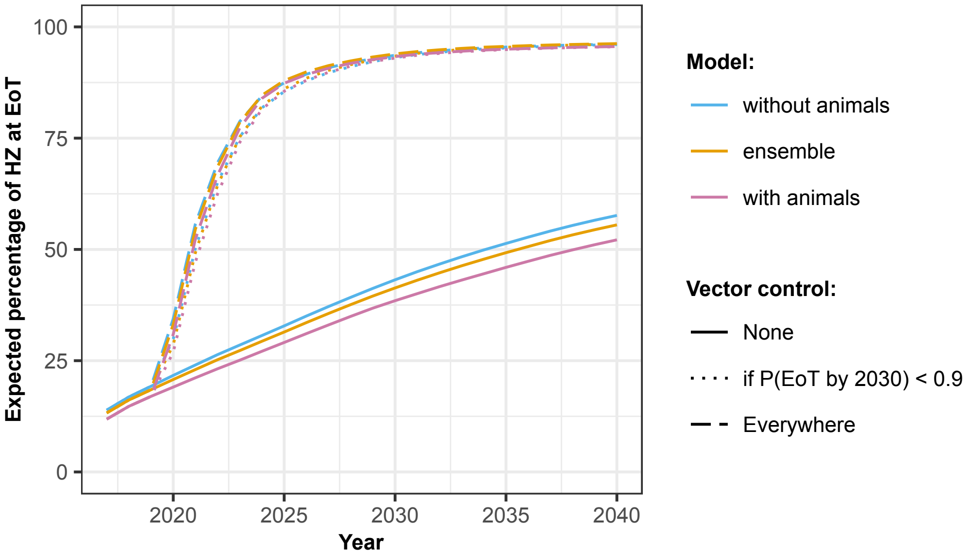

Fig D: The percentage of health zones studied expected to have reached elimination of transmission (EoT) against year, for the models without animal transmission, with animal transmission, and an ensemble of these two models. Active screening post-2016 was assumed to be at the mean level observed 2012–2016, and passive screening continued at the 2016 level of effectiveness. Vector control (VC) was either simulated in none (solid lines), all (dashed lines), or a subset of health zones in which the probability of reaching EoT by 2030 without VC was less than 0.9 (dotted lines; using this cut-off measure, VC was simulated in 77% of health zones in the ensemble model, 76% of health zones in the model without animal transmission, and in 79% of health zones in the model with animal transmission).

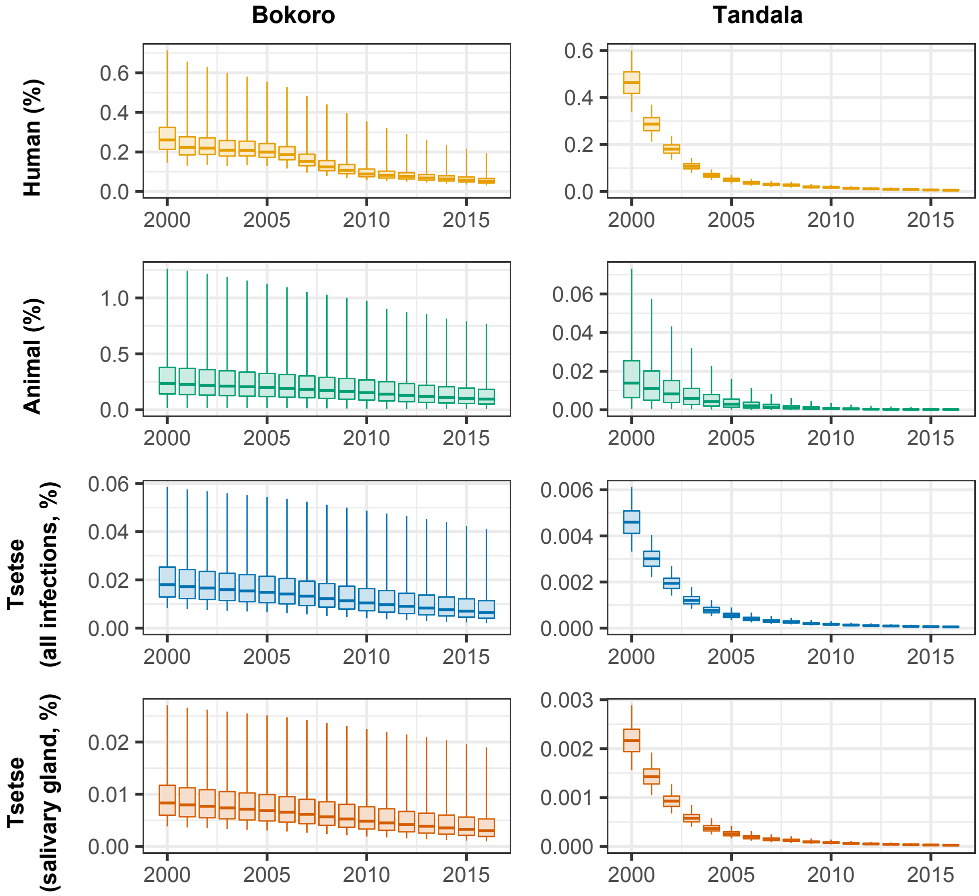

Fig E: Mean prevalence of infection within year as a percentage of the population. Human cases are considered across disease stages and population risk group. In vectors, the prevalence of all infections and infectious (salivary gland) infections is presented.

References

[1] M. S. Castaño, M. L. Ndeffo-Mbah, K. S. Rock, C. Palmer, E. Knock, E. M. Miaka, J. M. Ndung'u, S. Torr, P. Verlé, S. E. F. Spencer, and Others. Assessing the impact of aggregating disease stage data in model predictions of human African trypanosomiasis transmission and control activities in Bandundu province (DRC). *PLoS Neglected Tropical Diseases*, 14(1):e0007976, 2020.

[2] F. Checchi, J. A. N. Filipe, M. P. Barrett, and D. Chandramohan. The natural progression of *Gambiense* sleeping sickness: What is the evidence? *PLoS Neglected Tropical Diseases*, 2(12):e303, Dec. 2008.

[3] F. Checchi, F. Chappuis, U. Karunakara, G. Priotto, and D. Chandramohan. Accuracy of Five Algorithms to Diagnose *Gambiense* Human African Trypanosomiasis. *PLoS Neglected Tropical Diseases*, 5(7):e1233–15, July 2011.

[4] F. Checchi, S. Funk, D. Chandramohan, D. T. Haydon, and F. Chappuis. Updated estimate of the duration of the meningo-encephalitic stage in *gambiense* human African trypanosomiasis. *BMC Research Notes*, 8(1):292, 2015. ISSN 1756-0500. doi: 10.1186/s13104-015-1244-3.

[5] P.-H. Clausen, I. Adeyemi, B. Bauer, M. Breloeer, F. Salchow, and C. Staak. Host preferences of tsetse (Diptera: Glossinidae) based on bloodmeal identifications. *Medical and Veterinary Entomology*, 12(2): 169–180, Mar. 1998.

[6] F. Courtin, M. Camara, J.-B. Rayaisse, M. Kagbadouno, E. Dama, O. Camara, I. S. Traoré, J. Rouamba, M. Peylhard, M. B. Somda, M. Leno, M. J. Lehane, S. J. Torr, P. Solano, V. Jamonneau, and B. Bucheton. Reducing human-tsetse contact significantly enhances the efficacy of sleeping sickness active screening campaigns: a promising result in the context of elimination. *PLOS Neglected Tropical Diseases*, 9(8): 1–12, 08 2015.

[7] R. E. Crump, C.-I. Huang, E. S. Knock, S. E. F. Spencer, P. E. Brown, E. Mwamba Miaka, C. Shampa, M. J. Keeling, and K. S. Rock. Quantifying epidemiological drivers of *gambiense* human African Trypanosomiasis across the Democratic Republic of Congo. *PLOS Computational Biology*, 17(1):1–23, 01 2021. doi: 10.1371/journal.pcbi.1008532. URL https://doi.org/10.1371/journal.pcbi.1008532.

[8] S. Davis, S. Aksoy, and A. Galvani. A global sensitivity analysis for African sleeping sickness. *Parasitology*, 138(4):516–526, 2011.

[9] T. Hesterberg. Weighted average importance sampling and defensive mixture distributions. *Technometrics*, 37(2):185–194, 1995. ISSN 00401706. URL https://www.jstor.org/stable/1269620.

[10] C.-I. Huang, R. E. Crump, P. E. Brown, S. E. Spencer, E. M. Miaka, C. Shampa, M. J. Keeling, and K. S. Rock. Identifying regions for enhanced control of gambiense sleeping sickness in the democratic republic of congo. *Nature communications*, 13(1):1–11, 2022.

[11] C. Lumbala, P. P. Simarro, G. Cecchi, M. Paone, J. R. Franco, V. K. B. K. Mesu, J. Makabuza, A. Diarra, S. Chansy, G. Priotto, et al. Human African trypanosomiasis in the Democratic Republic of the Congo: disease distribution and risk. *International journal of health geographics*, 14(1):20, 2015.

[12] M. H. Mahamat, M. Peka, J.-b. Rayaisse, K. S. Rock, M. A. Toko, J. Darnas, G. M. Brahim, A. B. Alkatib, W. Yoni, I. Tirados, F. Courtin, S. P. C. Brand, C. Nersy, O. Alfaroukh, S. J. Torr, M. J. Lehane, and P. Solano. Adding tsetse control to medical activities contributes to decreasing transmission of sleeping sickness in the Mandoul focus (Chad). *PLoS Neglected Tropical Diseases*, 11(7):e0005792, 2017.

[13] A. Mpanya, D. Hendrickx, M. Vuna, A. Kanyinda, C. Lumbala, V. Tshilombo, P. Mitashi, O. Luboya, V. Kande, M. Boelaert, P. Lefèvre, and P. Lutumba. Should I get screened for sleeping sickness? A qualitative study in Kasai province, Democratic Republic of Congo. *PLOS Neglected Tropical Diseases*, 6(1):e1467, Jan. 2012.

[14] OCHA Office for the Coordination of Humanitarian Affairs. *Journees Nationales de Vaccination (JNV) Activities de vaccination supplementaire, RDC*, Accessed May 2016.

[15] S. Ravel, P. Grébaut, D. Cuisance, and G. Cuny. Monitoring the developmental status of *Trypanosoma brucei gambiense* in the tsetse fly by means of PCR analysis of anal and saliva drops. *Acta Tropica*, 88 (2):161–165, 2003.

[16] K. S. Rock, S. J. Torr, C. Lumbala, and M. J. Keeling. Quantitative evaluation of the strategy to eliminate human African trypanosomiasis in the DRC. *Parasites & Vectors*, 8(1):532, 2015.

[17] K. S. Rock, S. J. Torr, C. Lumbala, and M. J. Keeling. Predicting the impact of intervention strategies for sleeping sickness in two high-endemicity health zones of the Democratic Republic of Congo. *PLoS Neglected Tropical Diseases*, 11:e0005162, 2017.

[18] K. S. Rock, C.-I. Huang, R. E. Crump, P. R. Bessell, P. E. Brown, I. Tirados, P. Solano, M. Antillon, A. Picado, S. Mbainda, J. Darnas, E. H. Crowley, S. J. Torr, and M. Peka. Update of transmission modelling and projections of gambiense human african trypanosomiasis in the Mandoul focus, Chad. *Infectious Diseases of Poverty*, 11(1):11, 2022. doi: 10.1186/s40249-022-00934-8. URL https://doi.org/10.1186/s40249-022-00934-8.

[19] D. Rogers. A general model for the African trypanosomiases. *Parasitology*, 97(1):193–212, 1988.

[20] The World Bank. Data:Democratic Republic of Congo, 2015. URL https://data.worldbank.org/country/congo-dem-rep?view=chart. Accessed: 2015.

[21] I. Tirados, J. Esterhuizen, V. Kovacic, T. N. C. Mangwiro, G. A. Vale, I. Hastings, P. Solano, M. J. Lehane, and S. J. Torr. Tsetse control and gambian sleeping sickness; implications for control strategy. *PLoS Negl Trop Dis*, 9(8):e0003822, 2015. doi: 10.1371/journal.pntd.0003822.

[22] I. Tirados, A. Hope, R. Selby, F. Mpembele, E. M. Miaka, M. Boelaert, M. J. Lehane, S. J. Torr, and M. C. Stanton. Impact of tiny targets on glossina fuscipes quanzensis, the primary vector of human african trypanosomiasis in the democratic republic of the congo. *PLoS neglected tropical diseases*, 14 (10):e0008270, 2020.

[23] P. Touloupou, N. Alzahrani, P. Neal, S. E. F. Spencer, and T. J. McKinley. Efficient model comparison techniques for models requiring large scale data augmentation. *Bayesian Anal.*, 13(2):437–459, 06 2018. doi: 10.1214/17-BA1057. URL https://doi.org/10.1214/17-BA1057.

[24] United Nations Office for the Coordination of Humanitarian Affairs. The humanitarian data exchange, 2018. URL https://data.humdata.org/. (accessed: 06.08.2018).

[25] World Health Organization. Control and surveillance of human African trypanosomiasis: report of a WHO expert committee. Technical Report 984, World Health Organization, 2013.
