# Supplementary material for: Modelling to infer the role of animals in gambiense human African trypanosomiasis transmission and elimination in the DRC
Source: PLoS Negl Trop Dis. 2022 Jul 11;16(7):e0010599. doi: 10.1371/journal.pntd.0010599 (PMC9302778; doi:10.1371/journal.pntd.0010599)
Supplement: Attachment — Submitted filename: AnimalgHATmodel_Response_to_reviewers.pdf [file pntd.0010599.s003.pdf]

Response to Reviewers

Modelling to infer the role of animals in *gambiense* human African trypanosomiasis transmission and elimination in DRC

Ronald E Crump[1,2], Ching-I Huang[1,2], Simon E F Spencer[1,3], Paul E Brown[1,2], Chansy Shampa[4], Erick Mwamba Miaka[4], and Kat S Rock[1,2]

[1]Zeeman Institute for System Biology and Infectious Disease Epidemiology Research, The University of Warwick, Coventry, U.K.
[2]Mathematics Institute, The University of Warwick, Coventry, U.K.
[3]The Department of Statistics, The University of Warwick, Coventry, U.K.
[4]PNLTHA, Kinshasa, D.R.C.

May 27, 2022

The authors thank the four reviewers for the detailed comments on our manuscript. We have made numerous improvements to explanations in the text as a result. The reviewers' comments are reproduced in blue throughout, with our responses following where required.

Methods

Reviewer #1

The objectives of the study are clearly articulated with a clear testable hypothesis. I am not a mathematical modeller and am unable to comment critically on the modelling and statistical analyses; I presume that other reviewers will be able to do this. The models are based on previously published models produced by this research group and so on that basis I assume that the models etc are appropriate and correctly implemented.

No response required

Reviewer #2

Gambiense human African trypanosomiasis (gHAT) is still a major public health burden in the Democratic Republic of Congo. Although Trypanosoma brucei gambiense is believed to be transmitted primarily between humans due to Tsetse fly bits, the role of an animal reservoir in maintaining disease transmission is unclear.

The goal of this study was to look for evidence of gHAT transmission from an animal reservoir and consider how the existence of such reservoirs might effect efforts to eliminate transmission of gHAT in the DRC.

The main methods of this report were (1) formulation of deterministic models of gHAT transmission via the Tsetse fly, and (2) providing statistical support (in a Bayesian formalism) for the most likely models based on the actual public health data from the health zones of the DRC. Multiple models are constructed for individual health zones (choosing many parameters from a statistical distribution). The authors then calculated the Bayes support of models without animal transmission to models with animal transmission. The models employed are very similar to ones used previously by the authors, but in this report the authors are concerned in gauging the importance of possible animal reservoirs in blocking or slowing efforts to end transmission of gHAT in the DRC.

The model calculations suggests that in some health zones, animals do indeed serves as a reservoir of Trypanosoma brucei gambiense. In those zones, their models suggest that even a modest vector control effort wouldl contribute greatly to the goal of elimination of transmission of gHAT.

One could quibble about the details of specific parts of the human/tsetse/Trypanosoma system in their modeling schema, but the development of the models from real data and their evaluation and statistical weighing are impressive and thorough. The authors recognize in the final Discussion that non-deterministic models might be better in capturing the effects of stochastic fluctuations, but they argue that from previous experience, deterministic and stochastic models yield similar results, and that the use of fully stochastic models is unlikely to change the major conclusions that Tsetse control is needed to reduce transmission from local animals to humans.

No response required

Reviewer #3

The objectives are clearly articulated with a clear testable hypothesis, the design, population and analyses were appropriate, sample size sufficient, and ethical concerns were met.

Major Question 1: In the models, why does active screening only capture low-risk people? Maybe give a bit more of background on why the previous studies found this.

We agree we could provide more information and have added the text below to the methods:

This model structure has been fitted to a number of different data sets [R13, R12, R9, R6, R14], although certain aspects of the fitting procedure and simulation of passive surveillance have evolved since it was originally published. In Rock et al. [R13], Mahamat et al. [R9], and Rock et al. [R14] competing model structures were compared including those with possible participation of high-risk people in active screening. In these studies the model with random participation of both groups was not statistically supported by the longitudinal data as it was not able to capture the observed trends as well as models with systematic non-participation of high-risk groups. This aligns with anthropological evidence [R10] and programme experience which suggest certain groups (e.g. working age men) are often underrepresented in active screening activities.

Page 5 Point 3: How do CATT dilutions and video methods work to ID gHAT?

Point 3 has been modified to include some additional information:

. . . In former Orientale province pre-2013, Médecins Sans Frontières (MSF) were known to be operating an active screening algorithm based on CATT dilutions rather than parasitological confirmation. A positive CATT result at a dilution of 1:32 was declared to be a case and treated without parasitological confirmation. This is accounted for by modifying the parameters associated with the comparative algorithm for this period in this province: increasing the fixed sensitivity to 95%, and constraining the fitted specificity to be lower than that used outside this period. The use of video confirmation tools was introduced first in 2015 in Yasa Bonga and Mosango health zones in former Bandundu province and has subsequently been rolled out to the whole province. The microscopic parasitological confirmation procedure is video recorded and then reviewed centrally, to mitigate against false positive diagnoses. We simulate this as an increase to perfect specificity in 2018 for projections for other health zones in that province.

Page 5 Point 4: What is a Tiny Target?

The following has been added to Point 4:

. . . where Tiny Targets, *which consist of a square of insecticide-impregnated netting alongside a square of fabric in a shade of blue found to be highly attractive to tsetse*, have been used. . .

Page 5 Model Variants: It might be good to explain why the other models (1-3, 5-6, anything after 7?) were not selected?

We have added the following text:

Other model variants previously developed were not considered in this analysis as most have been shown to result in poor fits (without high/low risk structure and/or non-participation of high-risk people) [R13, R9, R14]. Two of those models are extensions of the models included in this study but with all combinations of participation/non-participation in active screening and risk possible. However these models are more complex (more parameters) and had similar statistical support under model fitting in past studies. Therefore we focus here on the slightly simpler variants.

Reviewer #4:

Major comments

Figure 1 Caption (and text elsewhere) suggests that model equations can be found in the supp, but I couldn't find them unless they meant Table S1.6 and S1.7. Can the equations or equivalently a fully annotated model diagram (i.e. with the rates of all state transitions) be provided in the supplementary materials please?

The equations have been added to the Supplementary Information.

S1.2 Birth rate: Can the authors justify using the fixed population size assumption? My understanding is that the population of DRC is growing rapidly (possibly >60% over the period covered by the data) and will also probably grow until 2040. I could imagine this could have some significant impacts on the data fitting for passive cases where the denominator is the *whole* population (with access to healthcare)

The model is internally scaled such that the population size in all years corresponds to the population in 2015. The birth rate also relates to this year.

Outputs are transformed to reflect population growth, with 3% annual growth assumed across DRC (regular, complete national census information which would allow localised differentiation is not available).

A footnote has been added to Table S1.2: "*The model is internally scaled such that the population size in all years corresponds to the population in 2015 (outputs are back-transformed to reflect an assumed annual population growth rate of 3% across DRC).*"

S1.2 Overdispersion for surveillance is fixed without a reference? How were these values chosen?

We added a reference to Crump *et al* (2021) for each of these overdispersion parameters, and added the following to the footer of Table S1.2:

"*Over-dispersion values were chosen based on a comparison of the median of the distributions of log posterior probability from MCMC runs with ρ fixed at a range of values for two example health zones under Model 4 [S7].*"

S1.5: What is an active/passive case (denoted A_{M1} and P_{M1})? How do these related to the compartments in Figure 1?

In section S1.5, observed annual case counts from data are denoted: A_{D1}, A_{D2}, A_{DU}, P_{D1}, P_{D2} and P_{DU}. These are cases from either active (A_{Di}) or passive (P_{Di}) screening, where $i \in \{1, 2, U\}$ indicate disease stage of 1, 2 or unknown (staging is not generally digitised in the data prior to 2015).

The equivalents to these from the model outputs are denoted A_{M1}, A_{M2}, P_{M1} and P_{M2}. Since we always know the stage (1 or 2) in the model simulations there is no requirement for a "U" (unknown stage) category for the model. These are computed using solutions to the ordinary differential equations for the given set of parameters aggregated across a year.

In terms of Figure 1, they relate to the transfer from infectious categories to the recovered category – the new annual reported case incidence. This is either by passive detection from stage 1 for year Y:

$$P_{M1}(Y) = \int_{Y}^{Y+1} \eta_H(Y)\big(I_{1H1}(t) + I_{1H4}(t)\big)\,\mathrm{d}t,$$

passive detection from stage 2

$$P_{M2}(Y) = \int_{Y}^{Y+1} (\gamma_H(Y) - \text{Death rate})\big((I_{2H1}(t) + I_{2H4}(t)\big)\,\mathrm{d}t,$$

or by active screening from the low-risk ($H1$) group in year Y

$$A_{M1}(Y) = z(Y)\text{Sens}I_{1H1}(Y) + z(Y)(1 - \text{Spec})\big(k_1 N_H - I_{1H1}(Y) - I_{2H1}(Y)\big)$$

and

$$A_{M2}(Y) = z(Y) \times \text{Sens} \times I_{2H1}(Y)$$

with variable active screening coverage by year, $z(Y)$ and fixed diagnostic sensitivity. A_{M1} also contains any false positives that may be been incorrectly identified from non-infected people based on the high but imperfect specificity of the active screening algorithm. We assume in DRC that all false positives would be assigned to be stage 1.

We have updated the SI to incorporate this information in the model fitting section.

How are the models initialised?

We added the following text in our SI (Now S1.1.5 "Running the models"):

Our ODE models were run assuming that, prior to 1998, they were at their endemic equilibrium. This endemic equilibrium was calculated analytically, based on model parameterisation, and used as an initial condition in the model code. In 1998 we assume active screening began at the same level reported in 2000 and that there was improvement to passive screening due to the availability of the CATT diagnostic test. In our model this has the effect of perturbing the dynamics away from endemic equilibrium and reducing transmission.

For fitting the models, there are two elements, each of which is initialised.

- *Two chains are run in the Markov Chain Monte Carlo (MCMC) used in the fitting. The chains are initialised using the fixed parameters and by random perturbations around supplied, individually valid, initial values of each parameter being fitted, rejecting those parameter sets that are do not produce a valid posterior probability.*
- *Each projection is initialised with a randomly sampled realisation from the posterior distribution of fitted parameters alongside the set of fixed parameters.*

The definition of EoT for the purposes of classifying model projections (less than 1 new case per year) needs to be stated earlier (I think this is first mentioned towards the end of the discussion). However the discussion of the limitations of using an ODE model in this way is handled very well!

Added the following to the description of Projections in the Methods:

Elimination of transmission was assumed to have been reached when the number of new human infections went below 1 per year. The probability of EoT by any year was the proportion of projections in which EoT had been reached by that year.

Minor comments

Line 111: Why was 13 datapoints chosen as the cutoff? Probably not worth a sensitivity analysis, but might be worth knowing why this cutoff was chosen. The text explains why it is more than 10 (in previous studies), but this doesn't really explain the choice of number.

The passage which talks about this has been expanded and separated as a standalone paragraph:

In Crump et al. *(2021), where the model without animal transmission was fitted, eight parameters were estimated in the adaptive MCMC fitting procedure in all health zones, and an additional four parameters were fitted [R6] in health zones where an improvement to passive screening was modelled (the former provinces of Bandundu and Bas Congo). For the model with animal transmission there are two additional parameters related to transmission to/from animal hosts that we choose to estimate within the model fitting. As a result, we chose to restrict the data to health zones containing at least 13 aggregated data points, that is either an active screening coverage of more than 20 people for the year or a non-zero number of cases detected by passive screening, this being 3 more aggregated data points than the 10 required in the earlier study with a slightly less complex model [R6]. Consequently we find a total of 158 health zones which we believe to have sufficient data for our analysis.*

Line 116-178: Seems out of place, i.e. should be in the limitation section of the discussion. I would expect the data subsection of the methods to describe the data that was used, with minimal commentary.

The information in these lines has been incorporated into the paragraphs which were found at lines 373–406 in the original manuscript:

...In order to consider a specific animal reservoir species, considerable data collection would be required to quantify the epidemiology of gHAT within the species and transmission between the animal species, tsetse and humans. In addition to prevalence data, data on relative abundance of different animal species in relation to human or tsetse density, and feeding proportions of tsetse on animals are key pieces of information which would required to understand the full picture of potential tsetse-animal transmission cycles. If a suite of potential animal reservoir species were considered this recording effort would increase dramatically, and even then it would be appropriate to consider a catch-all non-specific animal reservoir as well to pick up all the species that we had neglected to study. It is very difficult to imagine a scenario in which sufficient data collection across

time would allow resolution of differences between one or more specific potential animal reservoir species, and there has been very limited sampling of animal populations for gHAT globally [7].

Nevertheless, measures of location-specific prevalence of T. b. gambiense infection in animals could still provide valuable information for future modelling analysis, especially if very high prevalence or absence of infection is observed. At present there exists no 100% gambiense specific test to identify conclusively that a non-human animal has T. b. gambiense infection rather than other animal trypanosomes, which are generally circulating at a much higher prevalence. Meanwhile tsetse blood meal analyses could help better parameterise models, narrow our uncertainty, and identify animal species of interest for sampling. We would recommend that data from the same locations at coinciding times for both animals and humans would best enable improved model fitting, and caution that presence of non-specific Trypanosoma, or even T. brucei spp., unfortunately tells us little about transmission of the human-infective T. b. gambiense to and from animals. . .

Implicitly assumes that populations are well-mixed within districts. Smaller pockets can lead to difficulties in elimination efforts. This is a very reasonable assumption given the sparsity of data, but could be mentioned as a limitation. Might also be a alternative explanation to passive surveillance declines being slower than active surveillance

Added the following paragraph into the Discussion:

The geographical scale at which we have performed this study, the health zone level, is not an accurate representation of the distribution of either gHAT incidence or vector habitats. The model inherently assumes an equal mixing across the health zone. The presence of smaller pockets can lead to difficulty in achieving elimination but, when identified, also provide targeted sites for intervention activities.

S1.5.1 The way the likelihoods for stage 1 and 2 cases are split up are a little odd and took me a while to decide whether they make sense. A seperate BetaBin model for the observed number of A_1, A_2, P_1, P_2 cases would seem more natural to me. I'm guessing they formulated it this way because they didn't have the split by stage 1/stage 2 cases at every sample point and would simply drop the Bin model terms when this is not available. This section deserves a *little* more explanation.

You are correct that for many years we have no staging information, and in others not all reported cases are staged. We replaced the SI text *The Metropolis-Hastings MCMC used a log-likelihood function:* with

The log-likelihood function used in the adaptive Metropolis-Hastings MCMC contained two terms in each year for which reported case numbers were available for each source of reported cases (active or passive screening). These were:

- *a beta-binomial probability that the total number of cases reported in that year for that source came from the available population (either the reported number of people actively screened for active screening, or the health zone population for passive screening) with probability calculated from solving the ODE for the current set of parameters, and*
- *a binomial probability that the reported stage 1 cases comes from the total number of reported staged cases where the probability parameter again comes from the solution of the ODE. In many years staging is unknown and so this part of the log-likelihood will return zero and not contribute to our calculation. In some years, we only partially know staging information.*

This formulation allowed over-dispersion in the observed cases to be included, via the beta-binomial distribution, and any proportion of cases with reported disease stage to be appropriately accounted for (assuming that the reporting of staging information is independent of the disease stage). The log-likelihood function was as follows:

and the equation was updated slightly to include the cases detected by active or passive screening in the total number of cases reported for each screening type.

S1.4: Please provide details of how tsetse fly reduction is modelled here. This section was not really intelligible to me. Though I think it's fine to leave out *details* and refer readers to previous work, I believe that what's written in the paper and supp should be *intelligible* on its own. For instance, what does 'probability of both hitting a target and dying' mean? (After re-reading I'm guessing this refers to a tsetse fly hitting a lure/trap/device and dying, but at first I thought this was talking about public health elimination targets and was very confused!) What are f_T, t, and f_{max}? What is 'target efficacy' and why is it only around 0.03 and not .60 in the figures? Why does a 60%/90% tsetse reduction reduce tsetse numbers by more than 60%/90% in the figures (looks more like 75%/95% from the figures?) Is this to do with the timeframe over which the

interventions are applied? If in the *long-term* tsetse reductions will be much more than the stated 60%/90% then does it need to described differently (e.g. long-term average psuedo-equilibrium tsetse abundance reduce by X%)

The section on modelling the dynamics of tsetse populations was substantially modified to improve the clarity, including answering the range of senisible queries raised by the reviewer. We refer the reader to the revised SI - or the versions with changes highlighted - there being too many modifications within this section to include here.

S1.5.2 It's not clear what this imputation achieves, if anything. Surely imputing the failures with a NegBin to then use in the denominator of a Bin, nets no useful information for estimating parameters of interest θ? This is perhaps what they mean by 'canceling from the posterior', but I'm not sure what they are trying to say here. If it just drops out, why not just omit the data point? I'm familiar with imputation techniques for regression studies where there a few *covariates* missing and people don't want to throw away all the other non-missing covariate information; in these methods the approach is to use some information about patterns/associations within covariates to infer what the missing covariate might be. The (weak) analogy for the current study would be to use data on active surveillance denominators from other previous surveys in the focus (or other foci) to inform what the probable denominator might be for the focus with missing denominator. Though in principle I would expect this approach to provide some additional information about θ, I'm not suggesting they have to do something like this since it seems a lot of effort for not much gain. Also calling it a hierarchical prior is a bit odd! I would have thought that $A_D^-|\theta \sim NB(A_D(t), P^+(t))$ is the model and that (implicitly) there is only an improper uniform discrete prior for A_D^- over the integers here. Keeping the above as their model and adding an actual informative/empirical prior on A_D^- seems like a natural way to net more information on θ. I could have probably said this all more succinctly, but in summary, what the authors have done is probably harmless, but it might have some odd implications for the fitting if the imputation doesn't simply 'cancel out'.

High levels of active screening activity within a year will perturb the system and have knock on effects in subsequent years, for example reducing the number of undetected infected people and the length of time in which they can transmit the disease. The purpose of the imputation is to enable the inclusion of the number of cases reported from active screening in all years, even if the counts of the number of people screened was not available. By sampling the number of negative outcomes during active screening, we are able to reflect the changes to the system in all years and reflect our uncertainty about that through the sampling variation.

In S1.5.3, changed *To allow use of these records we impute the missing negative test results ($A_D^-(t)$) during the MCMC analysis* to read *To allow use of these records we impute the missing negative test results ($A_D^-(t)$) during the MCMC analysis, and this enables the model to account for perturbation of the system by unknown high levels of active screening activity within a year.* We also removed the word "hierarchical".

Results

Reviewer #1

The results match the analysis plan and are generally clearly presented.

I found the figures difficult to read - the colour scheme of the boxplots in figures 2 and 4-7 are particularly difficult.

We have recreated Figures 2, 6 and 7 with a modified, bolder colour scheme and a little extra space between the within-year pairs of boxplots to improve legibility.

Reviewer #2

Yes, the analysis presented matches the planned analysis. The figures in this paper and in the two supplemental files are well done and indicate both the geographic distribution of gHAT in the DRC health zones, and the results of their models. Figures 2 and 6 were especially informative in helping to visualize the implications of their model results.

No response required

Reviewer #3

The analysis matches the plan, results are clearly and completely presented, figures are clear.

Major Question 2: The authors find that animal involvement in maintaining transmission cycles is supported in Bokoro but not in Tandala. I noticed the two health zones have very different distributions of active testing, and I didn't see much discussion of this. Did the authors explore how the testing strategy/frequency/intensity could impact the results? Apologies if I missed this in the details somewhere.

Testing strategy, frequency and intensity are not independent of the prevalence of disease – the level of interventions will be adapted in response to the numbers of cases reported (and, at least in the short term, the number of cases reported reflects the intensity and efficiency of interventions) – therefore it is not possible to draw conclusions about the impact of the pattern of choices around intervention on the outcome of modelling to detect contribution of animals to transmission.

In Bokoro, there is a slow decline in case reporting despite on-going high levels of active screening and improvement over time to passive detection rates. This slow decline in Bokoro probably provides some evidence for a contribution of animals to transmission.

Added to the discussion:

Our ability to explain the levels of statistical support for either model studied here in terms of the historical data and in particular past interventions is constrained by the intimate connection between interventions – in particular the frequency and scale of active screening – and case detections. This difficulty is exacerbated by the, unknown to us, impact of geographically variable levels of potential animal animal host species and habitat, tsetse population and habitat and proximity/overlap between all host and vector habitats. Two groups of health zones where we might have expected to find statistical support for the model with animal transmission can be thought about. Firstly, those where interventions have brought about a reduction in case reporting to almost none and a relatively lengthy timeseries of low-level case reporting exists subsequent to this. This would be the case for health zones in Equateur, where high levels of AS activity in the early 2000s resulted in rapid reductions in the number of cases being reported followed by low-levels of case reporting detected by passive and active screening in later years. Secondly, health zones such as Bokoro where the decline in case reporting is slower than might be expected given the on-going high levels of annual AS coverage.

Reviewer #4

The results here match the methods and provide a mostly clear picture of the results. There a number of minor presentation points which I have included in a later section. However, a couple points of more considerable concern are listed here:

Lines 277-279: "Both Model 4 and Model 7 fit very similarly to the observed data points. However in Bokoro health zone, which has strong support for the model with animal transmission..." These two statements appear rather contradictory. Please clarify

We agree this was confusing. We have now reworded to:

Both the models with and without animal transmission appear visually to have similar fits to the observed data points. However in Bokoro health zone, there is stronger statistical support for the model with animal transmission, there are more new human infections each year, and also more unreported deaths when animal transmission is included (deaths not shown in the figure)

Fig 7: The sharp drop-off (2017-2018) then slow decline (2019-2040) of predicted actively identified cases warrants explanation. Almost looks like a bug!

Thanks for flagging the missing explanation. We have added the following text to the caption:

In Bokoro, and other health zones in the former province of Bandundu, the specificity of active screening was assumed to increase to 100% in 2018 due to video confirmation.

and the following in the methods section:

Following the introduction of video confirmation of active case parasitology across former Bandundu province in 2018 we assume that the specificity of the active screening algorithm is improved to 100% at this point and consequently there is a drop in active case numbers. This is particularly noticeable for our projections in Bokoro health zone.

Conclusions

Reviewer #1

The conclusions are supported by the data presented but I felt there were disparities in tone between the Abstract/Author summary and the Discussion. In particular:-

The Abstract reports moderate to high evidence of statistical support for some animal transmission. Author summary reports 'statistical evidence' for animal transmission - ie nothing to qualify the strength of evidence Discussion reports 24 health sones with 'substantial to decisive levels of statistical support'.

I think a consistent and conservative ('substantial to decisive') conclusion about the role of animals would be welcome across all sections and, if space allows, some indication of how the 'substantial to decisive' conclusion is made.

The statistical support for the model with animal transmission is substantial to decisive in only 24 of 158 health zones studied, conversely the statistical support for the model without animal transmission is substantial to decisive in 71 of these 158 health zones. We believe that if animals do contribute to gHAT transmission then in the future – in the absence of interventions which impact on transmission in animals directly as well as humans – longer time series of observed numbers of new cases with persistent, on-going case detection at low levels would be likely to increase the number of health zones in which there is support for the model with animal transmission. However, placing greater emphasis on the level of support found in this small minority of health zones on the basis of this hypothesis would not constitute a conservative conclusion.

I also found that important conclusions in the Discussion did not find their way to the Abstract. For instance, the conclusion that 'it is extremely unlikely that a maintenance animal reservoir exists in DRC' is an important finding for policy makers and therefore should be in the Abstract. My understanding is that the authors conclude that animal reservoirs are not preventing elimination of transmission but may delay its achievement.

We have slightly modified the paragraph on conclusions in the abstract, however we feel that the conclusion you refer to was already included in the text albeit phrased differently because maintenance reservoirs have not been defined in the abstract.

We conclude that there are 24/158 health zones where there is substantial to decisive statistical support for some animal transmission. However – even in these regions – **we estimate that animals would be extremely unlikely to maintain transmission on their own**. *Animal transmission could hamper progress towards EoT in some settings, with projections under continuing interventions indicating that the number of health zones expected to achieve EoT by 2030 reduces from 68/158 to 61/158 if animal transmission is included in the model. With supplementary vector control (at a modest 60% tsetse reduction) added to medical screening and treatment interventions, the predicted number of health zones meeting the goal increases to 147/158 for the model including animal transmission. This is due to the impact of vector reduction on transmission to and from all hosts.*

I think the Discussion could merit some more discussion of the relevant literature and the practical implications of their work. For instance, some of the conclusions that I think might emerge from such discussion are:-

It is practically impossible to sample sufficient number of potential animal hosts to test the hypothesis that animals are acting as reservoirs. This seems to be the implication of the sentence the 'prevalence in animals is even lower than that found in humans'. If prevelance in humans is approaching $1/10,000$ then presumably the predicted prevalence in animals is $< 1/10,000$. Expanding on this would guide researchers and funders concerned with identifying animal reservoirs empirically.

In supplemental figure S1.5 we show the expected prevalence for animals during the data period (2000–2016) in the case of there being some transmission from animals in each of our example health zones. In Bokoro, where there is more support for animal transmission, prevalence in animals is "relatively high" with similar expected magnitude of human and animal prevalence (still less than 0.1% for later years). In Tandala there is little support for animal transmission and we expect virtually zero prevalence in animals.

We have added the following text in the discussion:

The average within-year prevalence estimated from the model outcomes is presented in the SI (S1 Materials and Methods, Fig S1.5) for humans, animals and tsetse in Bokoro and Tandala health zones, confirming that the prevalence in animals could be even lower than that found in humans. In Bokoro – where there is substantial statistical support for the animal transmission model – we estimated that at the end of the data

period (2016) we would expect to have an infection prevalence of <0.1% in humans and a similar expected magnitude in animals. This value suggests that thousands of animal samples might have to be collected and analysed to have a moderate probability of finding possible animal infection with T. b. gambiense, and absence of infection in these samples would not rule out some animal transmission at even lower prevalences.

The stronger evidence for animals being reservoir hosts was related to certain health zones which, I think, were also places with low case incidence following intensive active screening. The authors speculate that this is likely to occur in other health zones as they move to this low incidence scenario. Do the authors think that this the role of animal reservoirs is obscured in high incidence settings - rather like guinea worm - or is this an artefact of the models? Are there other hypotheses to explain the difference between Health Zones? For instance, do parts of Equateur have higher densities of livestock?

We agree that many health zones where there is strong to decisive support for the animal model are those where case reporting from humans is now very low, but has been low for some time. Like Guinea worm, we expect any impact of possible animal transmission to be most apparent as we approach zero.

We believe the existing sentence below in the beginning of our discussion covers this first part:

In many of these health zones we identified a similar trend in the data that the health zones with support for the model with animal transmission are among those with a relatively long time series of low-level case detection by passive screening while also having been subject to reasonable active screening coverage, if only historically.

We also added the following:

...there would be more support for the presence of an animal reservoir contributing to transmission of gHAT as more data become available. As with Guinea worm, signal from potential animal transmission should be most noticeable as we approach zero.

However we also note that there are some "weak support for animals" health zones in higher prevalence areas where case reporting has not fallen as rapidly as we might hope/expect with sustained high coverage active screening (e.g. in former Bandundu province). So we added:

Some health zones with moderate case reporting in 2016 have weak to substantial support for animal infection. Kwamouth, Bagata, Mokala and Bokoro health zones – all located in former Bandundu province – have had steady but relatively slow reductions in human gHAT reporting over the data period despite persistent, high-coverage active screening. This could be a early (although inconclusive) signal that interventions may be being hindered.

Several countries appear to have achieved or be approaching the elimination of transmission (e.g. Mali, Burkina Faso, Cote d'Ivoire, Uganda). Does this fit with the notion that there are animal reservoir hosts for gHAT. Do the authors think that DRC is different? Could this be a contribution to the high disease burden?

Added to discussion:

Between-location variation in factors that may be associated with animal transmission of gHAT; such as the animal species available, abundance of these species, tsetse density, tsetse feeding preferences and the size and proximity of the host and vector habitats, will all have an impact on the contribution of animals to transmission in a location. They would also be expected to impact on the ability of modelling of human case numbers to detect animal transmission in any given location. The success of human medical interventions would imply that any contribution of animals to transmission is not huge. However, the slow decline in cases in Bokoro health zone despite on-going high levels of coverage by active screening (see Fig 2) may be indicative of a higher contribution. As evidenced by the very small number of cases being reported in, for example, Côte d'Ivoire, Uganda, Mali and Burkina Faso [R15] the contribution of animals to transmission in these locations is likely low, if any. However, this may not indicate an inherent biological difference between DRC and other countries but merely reflect that there is a great variety in the animal/tsetse abundance and tsetse feeding preferences, which we also believe to be reflected in the range of results across the health zones of DRC.

Some discussion about published empirical evidence for animal reservoirs being important would strengthen the discussion.

We added some additional text and a reference in the discussion to point readers towards the more comprehensive review of empirical evidence:

...there has been very limited sampling of animal populations for gHAT globally [R2]

In the Abstract, I found the use of numbers of health zones with evidence of some animal transmission (24/158) followed by EoT being reduced from 68 to 61 Health Zones if animals are reservoir hosts, and then back to 147/158 achieving elimination with the addition of vector control rather confusing. I thing it would help to use the same denominator (158) so the reader can judge the importance of the finding and proposed solution.

Changed 68 and 61 to 68/158 and 61/158.

Reviewer #2

The data presented are the results of their modeling, and do support their conclusions that (1) some of the health zones in the DRC do have gHAT reservoirs and (2) some effort is needed to control the Tsetse fly vector in those health zones in order to reduce gHAT transmission. These conclusions are of the upmost importance in guiding efforts to control or eliminate gHAT in the DRC.

Reviewer #3

Conclusions supported by the data, limitations clearly described, discussion of insight provided was great, and public health relevance was clear.

Reviewer #4

I believe the conclusions are mostly very sound and that most of the main limitations are clearly stated with one main exception.

Throughout the paper, the paradigm is that of three kinds of vertebrate hosts, 'low risk humans (actively and passively screened)', 'high risk humans (only passively screened)' and 'other animals'. However, it appears to me that a model that replaces the 'other animals' with a third class of humans who are not actively or passively screened might have a similar model form and very similar model predictions/likelihood (the main difference would be the life expectancy of the hosts, but I'd be surprised if this makes much difference to the model fit). This is not a problem in the construction of the models per se (they appear to have done an excellent job of working with the available data!), but I think it means that the results need to be interpreted a little more cautiously. For instance, health districts with 'strong evidence' for the animal models might equally be districts with a human subpopulation that isn't captured (or very poorly captured) by active/passive surveillance (e.g. certain groups defined by access to healthcare, geography, age, sex, disability, social standing, individual behaviour etc.). This probably doesn't affect the predictions about time to EoT and impact of tsetse reductions, but looking for an animal host might prove a wild goose chase! I'm not suggesting they need to fit or consider and alternative model, but something along these lines might need to be raised in the discussion.

We agree that alternative possibilities should be discussed and, as such, have added the following in the discussion:

In the present study we have focussed on transmission models with high- and low-risk people with or without additional animal transmission. In our models we assume that some people may systematically not participate in active screening activities but that they would be expected to either be eventually be detected and treated through passive screening or die (possibly undetected) from gHAT disease. In regions where we find more statistical support for the model with animal transmission, it could be possible that alternative model variants could also generate similar patterns in observed case dynamics. One hypothesis is that undetected asymptomatic human infections with long-term asymptomatic carriage and ability to (potentially) self-cure could contribute to sustained transmission of gHAT [R2, R3].

The models variants in the present paper already allow for long-term parasite carriage for a small proportion of individuals via their exponentially distributed infection periods, however not for self-cure or for skin-only parasite infection which would be missed in current screening algorithms. A few models considering potential self-curing asymptomatic transmission have been previously developed [R5, R4, R1] and show that such a reservoir could hinder progress towards EoT, however, as with animal data, data on frequency and duration of such infections is sparse. Future modelling work should assess whether or not it is possible to distinguish signals in routinely reported gHAT case data which support animal transmission over asymptomatic transmission, or whether a mix of other types of human and/or non-human animal data would be needed to make this distinction.

Editorial and Data Presentation Modifications?

Reviewer #1

The article is well produced and written.

Reviewer #2

The only criticism I have is that the authors state in the "Data Availability" statement that "No - some restrictions will apply". They then state "The gambiense human African trypanosomiasis (HAT) data were obtained from the WHO HAT Atlas and are subject to a data sharing agreement. Interested parties should apply to the WHO in order to gain access to these data."

I have some qualms about this restriction, especially as the credibility of the WHO has come under attack because of the fraught relationship between the WHO and China over the origins of SARS-CoV-2. I state upfront that I think those attacks are mostly unfair to WHO, but then, in the case of gHAT, what is the criteria for an "interested party" to gain access to the data? Is patient confidentiality the main reason for the restriction? It could be that PLoS and the authors have already negotiated an agreement about the restricted access based on legal and ethical concerns, but I have no information about that.

The authors are subject to a negotiated data sharing agreement and are unable to provide access to the data to third parties. Full details are provided so that third parties may request the data and all requests will be reviewed by the WHO committee to determine whether planned use of the data is acceptable.

Reviewer #3

Page 2 line 29: realized → "achieved" is a better word here.

The suggested revision has been made.

Page 2 line 29: also, "historically assumed" - is there a reference for this? Do the authors mean the models have only ever just considered human-to-human transmission?

We have slightly amended our text here to say:

gHAT is a vector-borne infection, historically assumed by the infectious disease community to be primarily transmitted to and from humans by blood-feeding tsetse [R7]

This has not simply been a modelling assumption to reduce complexity, but previously it was a wide-spread notion in the HAT community that animals do not play a large role (if any) in gHAT transmission dynamics, unlike it's sister disease *rhodiesense* HAT.

Page 2 line 39: directly are → directly is

The suggested revision has been made.

Page 3 line 58-59: cite these studies here, either after this clause, or at the end of the sentence.

Citations added [R8, R13, R11, R9, R14].

Page 3 line 66: already defined DRC, so just use DRC.

The suggested revision has been made.

Page 3 line 79: build upon this prior work, using. . .

The suggested revision has been made.

Page 5 line 188: assume, not believe

The suggested revision has been made.

Page 7 line 242: great tool!

Thank you.

Page 8 Figure 3 caption: why specify " ' Barely worth mentioning' "? Just say "Weak". Let the reader decide the worth of that result.

‘Barely worth mentioning’ is the description used by Jeffreys when defining that categorisation of Bayes factors. We used Weak in most scenarios for brevity, but believe Jeffreys’ nomenclature to be better descriptive of the value of such a finding. However, the suggested revision has been made.

Page 11 Line 394 - yes!!!

Reviewer #4

Line 131 and 143 (and elsewhere): Reusing Model numbers from previous work is confusing. If the name being reused was informative, then reuse would be fine. I think it would be clearer to give them informative names (for instance something like: Model 4 $\longrightarrow$ anthroponotic model) or new uninformative names (Model A, Model B...). Then at key points they could link back to previous work (e.g. “Model X is equivalent to Model 4 from previous work”)

We have removed references to Model 4 and Model 7 throughout the manuscript and supplementary information, except were we introduce them in the context of previous work. We have replaced them with *the model without animal transmission* and *the model with animal transmission*, respectively, or *the models with and without animal transmission*.

Figure 1: Green diamond say non-reservoir animals. Should this be non-human animals? The question is whether or not they are reservoirs, no?

This has been changed to read *animal, no transmission*.

Figure 1: No dotted (transmission) lines to or from H_4 compartments (though from Table S1.6 I gather there is transmission to/from H_4.

These lines have been added.

Figure 1: Adding legend/text describing what the different lines (dotted/solid) mean which might help non-modelling readers.

The following sentence has been added to the caption for Figure 1: *Solid lines represent transition within a pathway, while broken lines represent transmission.*

Line 156 (and elsewhere): 'Passive detection rates'. I assume here the authors mean the probability of detection through passive surveillance and not simply the incidence of cases reported by passive detection? Some authors insist that the word 'rate' be reserved to talk the frequency of events per unit time (e.g. death rate due to cancer would mean the number of people dying due to cancer per year/month/etc., not the proportion of cancer patients who die from the disease) . I think this is good practice in general (though I try not to be pedantic) but using the term rate here (and elsewhere?) is genuinely confusing! There is also a missing 'and' or fullstop in line 156.

We agree that the word “rate” should not be used interchangeably with “probability”, however in our manuscript we confirm we do mean rate. To clarify to other readers we added an extra note for clarity:

Passive detection rates can increase over time so that infected individuals are detected and treated more rapidly now than in the past.

Furthermore, in our model of passive detection we do assume that the proportion of individuals surviving gHAT infection also increases as the rates increase (see new details in the SI, section S1.3).

Changed *“Passive detection rates can increase over time, we simulate this...”* to *“Passive detection rates can increase over time so that infected individuals are detected and treated more rapidly now than in the past. We simulate this...”* on line 156.

S1.6: Side by side, $\mathbb{1}^n$ and $\mathbb{0}^n$ suggest blocks of ones and zeros respectively which is confusing. I'm guessing the $\mathbb{1}^n$ in the equations are meant to be identity matrices (usually notated I_n) rather than matrices of all ones (usually notated J_n)? Also this whole section might be more easily summarised in terms of sub-matrices of K.

The matrix definitions in section S1.6 have been rewritten in terms of $\mathbf{I}_n$ and $\mathbf{0}_{n,m}$ submatrices. The bold notation for matrices has been kept to minimise the possibility of confusion with other italic uppercase notation used in the models.

Fig 2 and 6: The y-axis labels need to be clarified. After lots of reading between the lines I think they mean (from top to bottom) Number of people actively screened per year, Number of cases reported from active screening per year, number of cases reported from passive screening per year and number of new infections per year. I think the confusion was because 'active cases' usually means 'the number of people who are known to infected right now'. Perhaps 'actively detected cases' and 'passively detected cases' would clarify this. A similar change throughout the paper might be helpful. The cases themselves are not 'active' or 'passive'!

The y-axis labels have been modifed as suggested to read 'Actively screened', 'Actively detected cases', 'Passively detected cases' and 'New human infections'.

Fig 2 and 6: It might also be helpful to indicate the populations of the two health districts to get a sense of proportion.

'2015 population estimate: XX', where XX = 218248 or 274944 for Bokoro and Tandala, respectively, added as subheadings below health zone names in these figures.

Line 329: What is the MeanAS strategy?

Apologies. We use that abbreviation widely in other studies where we have more future intervention strategies, and it was re-used here in error.

The text . . . *under the MeanAS strategy. . .* has been replaced by . . . *under the assumption that active screening continues with the average annual number of people screened in the period 2012–2016 ($48\,630$ in Bokoro) being screened each year and without vector control. . .*

Line 61/62: I had to re-read this a few times – I think the paragraph break threw me off by making it unclear what 'these studies' (line 62) referred to.

Removed the paragraph break.

Line 72: Perhaps better if they use the phrase 'maintenance reservoirs' here and throughout. It's introduced above and used again to state the goals of the study

A number of changes have been made throughout the manuscript and SI in response to this. Please refer to the versions of the documents with changes highlighted to more easily find these.

Lines 82-84: 'If there is animal transmission' – this qualifier is unnecessary. They are looking at trends for both kinds of models. Perhaps they meant something like: Based on current trends, what will projections to 2030 and beyond look like and what role does animal transmission play in this (if any)?

The suggested revision has been made.

Fig 3: The caption is overly technical with the regards to Bayes factors. The description doesn't help people who don't understand Bayes Factors or people who understand Bayes Factors (who just need to know the cutoffs). I suggest dropping everything in parentheses and replace all the K_{ij} with 'BF' (or some other shortening for Bayes factor)

The caption has been revised. . .

Support for model either with or without animals contributing to transmission of gHAT. Levels of support taken from whichever of the two Bayes Factors (BF, with either evidence for the model with or without animal transmission as the denominator) exceeded 1. Weak, $10^0 < BF < 10^{\frac{1}{2}}$*; Substantial,* $10^{\frac{1}{2}} < BF < 10^1$*; Strong,* $10^1 < BF < 10^{\frac{3}{2}}$*; Very Strong,* $10^{\frac{3}{2}} < BF < 10^2$*; and Decisive,* $BF > 10^2$*. . . .*

Line 323: phrase 'at this level' is redundant?

Moved 'at this level' to refer specifically to continuing active screening at the level described in the preceding sentence. . .

. . . continuing active screening at this level, alongside passive screening at the current level of effectiveness, from 2017 to 2040. . .

Line 360: 'observed data timeseries grows' sounds like they are talking about growing incidence, though I'm guessing they mean 'as more data becomes available'

The suggested revision has been made.

Summary and General Comments

Reviewer #1

(No Response)

Reviewer #2

As stated above, the efforts to develop realistic models of gHAT transmission and provide good statistical support for them are impressive and thorough. The authors are concerned about using their results to guide real-world efforts to control gHAT in a country plagued by this disease, and I think this paper gives good support for the need to control the Tsetse fly vector.

This reviewer wishes that the authors could have had space to present some stochastic models. But perhaps that needs a more theoretical paper than this one, which is clearly aimed at influencing the implementation of needed public health measures. As the discussion and results stand now, this manuscript is complete (aside from the data access issue mentioned in the Editorial and Data Presentation Modifications section).

Thank you for your helpful review. The authors recognise your concerns over data accessibility, however this is beyond our control.

Reviewer #3

The current article "Modelling to infer the role of animals in gambiense human African trypanosomiasis transmission and elimination in DRC" by Ronald E Crump and colleagues is a well-written and important contribution to the fight against an NTD that can circulate in both human and non-human hosts. This sort of one-health modeling approach is necessary for a realistic estimation of the capacity of EoT programs to achieve their goals. The methods appear rigorous, claims are supported by the results shown, and this study makes a valuable contribution to the field with clear implications for public health.

I have some questions for the authors, though. See above.

Thank you for your thorough review. We hope that the responses and modifications outlined earlier in this document have addressed these questions.

Reviewer #4

This is in my opinion a very interesting and well executed study. The authors have done an excellent job of working with the available data. The introduction lays out the complex possible relationships that could exist between multiple reservoirs and the rest of the paper continues with this clarity of purpose and outcomes. Their use of interactive data visualisation and maps is excellent. Though I have identified an unfortunately long list of questions and issues, they are all individually pretty minor and mostly around presentation and interpretation. I do not think any further experiments are necessarily needed (unless the odd outcomes in Figure 7 turn out to be a real bug!). I have no concerns about dual publication, research ethics, or publication ethics

Thank you for your thorough review. We hope that we have addressed your concerns through the responses and modifications outlined earlier in this document. Just to reiterate that the sudden dip in cases from active screening in 2018 for Bokoro health zone resulted from increasing the specificity of the diagnostic algorithm to 100% in response to the introduction of video review/confirmation of microscopic parasitological identification of the parasite across the whole of the former province of Bandundu.

References

[R1] M. Aliee, M. J. Keeling, and K. S. Rock. Modelling to explore the potential impact of asymptomatic human infections on transmission and dynamics of african sleeping sickness. *PLoS computational biology*, 17(9):e1009367, 2021.

[R2] P. Büscher, J.-M. Bart, M. Boelaert, B. Bucheton, G. Cecchi, N. Chitnis, D. Courtin, L. M. Figueiredo, J.-R. Franco, P. Grébaut, E. Hasker, H. Ilboudo, V. Jamonneau, M. Koffi, V. Lejon, A. MacLeod, J. Masumu, E. Matovu, R. Mattioli, H. Noyes, A. Picado, K. S. Rock, B. Rotureau, G. Simo, S. Thévenon,

S. Trindade, P. Truc, and N. V. Reet. Do cryptic reservoirs threaten *gambiense*-sleeping sickness elimination? *Trends in Parasitology*, 34(3):197–207, 2018.

[R3] P. Capewell, C. Cren-Travaillé, F. Marchesi, P. Johnston, C. Clucas, R. A. Benson, T.-A. Gorman, E. Calvo-Alvarez, A. Crouzols, G. Jouvion, et al. The skin is a significant but overlooked anatomical reservoir for vector-borne african trypanosomes. *Elife*, 5:e17716, 2016.

[R4] P. Capewell, K. Atkins, W. Weir, V. Jamonneau, M. Camara, C. Clucas, N.-R. K. Swar, D. M. Ngoyi, B. Rotureau, P. Garside, A. P. Galvani, B. Bucheton, and A. MacLeod. Resolving the apparent transmission paradox of African sleeping sickness. *PLOS Biology*, 17:1–6, 01 2019.

[R5] M. S. Castaño, M. L. Ndeffo-Mbah, K. S. Rock, C. Palmer, E. Knock, E. M. Miaka, J. M. Ndung'u, S. Torr, P. Verlé, S. E. F. Spencer, and Others. Assessing the impact of aggregating disease stage data in model predictions of human African trypanosomiasis transmission and control activities in Bandundu province (DRC). *PLoS Neglected Tropical Diseases*, 14(1):e0007976, 2020.

[R6] R. E. Crump, C.-I. Huang, E. S. Knock, S. E. F. Spencer, P. E. Brown, E. Mwamba Miaka, C. Shampa, M. J. Keeling, and K. S. Rock. Quantifying epidemiological drivers of *gambiense* human African Trypanosomiasis across the Democratic Republic of Congo. *PLOS Computational Biology*, 17(1):1–23, 01 2021. doi: 10.1371/journal.pcbi.1008532. URL https://doi.org/10.1371/journal.pcbi.1008532.

[R7] J. R. Franco, P. P. Simarro, A. Diarra, and J. G. Jannin. Epidemiology of human African trypanosomiasis. *Clinical epidemiology*, 6:257, 2014.

[R8] S. Funk, H. Nishiura, H. Heesterbeek, W. J. Edmunds, and F. Checchi. Identifying transmission cycles at the human-animal interface: The role of animal reservoirs in maintaining *Gambiense* human African trypanosomiasis. *PLOS Computational Biology*, 9(1):1–9, 01 2013. doi: 10.1371/journal.pcbi.1002855. URL https://doi.org/10.1371/journal.pcbi.1002855.

[R9] M. H. Mahamat, M. Peka, J.-b. Rayaisse, K. S. Rock, M. A. Toko, J. Darnas, G. M. Brahim, A. B. Alkatib, W. Yoni, I. Tirados, F. Courtin, S. P. C. Brand, C. Nersy, O. Alfaroukh, S. J. Torr, M. J. Lehane, and P. Solano. Adding tsetse control to medical activities contributes to decreasing transmission of sleeping sickness in the Mandoul focus (Chad). *PLoS Neglected Tropical Diseases*, 11(7):e0005792, 2017.

[R10] A. Mpanya, D. Hendrickx, M. Vuna, A. Kanyinda, C. Lumbala, V. Tshilombo, P. Mitashi, O. Luboya, V. Kande, M. Boelaert, P. Lefèvre, and P. Lutumba. Should I get screened for sleeping sickness? A qualitative study in Kasai province, Democratic Republic of Congo. *PLOS Neglected Tropical Diseases*, 6(1):e1467, Jan. 2012.

[R11] A. Pandey, K. E. Atkins, B. Bucheton, M. Camara, S. Aksoy, A. P. Galvani, and M. L. Ndeffo-Mbah. Evaluating long-term effectiveness of sleeping sickness control measures in Guinea. *Parasites & Vectors*, 8(1):550, 2015.

[R12] K. Rock, A. Pandey, M. Ndeffo-Mbah, K. Atkins, C. Lumbala, A. Galvani, and M. Keeling. Data-driven models to predict the elimination of sleeping sickness in former Equateur province of DRC. *Epidemics*, 18:101–112, 2017.

[R13] K. S. Rock, S. J. Torr, C. Lumbala, and M. J. Keeling. Quantitative evaluation of the strategy to eliminate human African trypanosomiasis in the DRC. *Parasites & Vectors*, 8(1):532, 2015.

[R14] K. S. Rock, C.-I. Huang, R. E. Crump, P. R. Bessell, P. E. Brown, I. Tirados, P. Solano, M. Antillon, A. Picado, S. Mbainda, J. Darnas, E. H. Crowley, S. J. Torr, and M. Peka. Update of transmission modelling and projections of gambiense human african trypanosomiasis in the Mandoul focus, Chad. *Infectious Diseases of Poverty*, 11(1):11, 2022. doi: 10.1186/s40249-022-00934-8. URL https://doi.org/10.1186/s40249-022-00934-8.

[R15] World Health Organisation. Number of new reported cases of human african trypanosomiasis (*T.b. gambiense*), 2021. URL https://www.who.int/data/gho/data/indicators/indicator-details/GHO/hat-tb-gambiense. (accessed: 01.03.2022).
